# Supplementary material for: Post-translational modifications via serine/threonine phosphorylation and GpsB in Streptococcus mutans
Source: mSystems. 2025 Oct 30;10(11):e01105-25. doi: 10.1128/msystems.01105-25 (PMC12625713; doi:10.1128/msystems.01105-25)
Supplement: Supplemental Material — Fig. S1 and S2; Tables S1-S11. [file msystems.01105-25-s0001.pdf]

Supplemental Material for:

Title: Post-translational modifications via serine/threonine phosphorylation and GpsB in *Streptococcus mutans*

Authors: Sangam Chudal[1], Courtney Dover[1], Tiffany Haydt[1,2], Shawn M. King[1], Robert C. Shields[1,3]*

Affiliations:
[1]Department of Biological Sciences, Beck College of Sciences and Mathematics, Arkansas State University, Jonesboro, Arkansas, USA
[2]Department of Asian and Middle Eastern Studies, College of Arts and Sciences, University of North Carolina at Chapel Hill, Chapel Hill, North Carolina, USA
[3]Department of Oral Biology, College of Dentistry, University of Florida, Gainesville, Florida, USA

*Address correspondence to: rshields@dental.ufl.edu

This file includes:
Figs. S1 – S2
Table S1 – S11

```
CLUSTAL O(1.2.4) multiple sequence alignment


Sp      ----masiiifsakdifeqefgre-vrgynkvevdeflddvikdyetyaalvkslrqeiad 55
Sm      ----MASIMYTPKDIFEQEFKSS-MRGYDKKEVDEFLDDIIKDYETYISTIEELRQENTR 55
Ef      ----manlvyspkdilqkefktkmmngydpievdefldnvikdyeaynkellslqeensr 56
Lm      mtseqfeyhltgkeilekefktg-lrgyspedvdefldmvikdystftqeiealqaenir 59
Bs      --mladkvklsakeilekefktg-vrgykqedvdkfldmiikdyetfhqeieelqqenlq 57
              .   : *:*:::**    :.**.  :**:*** :****.::   :  *: *

Sp      lkeeltrkpkpspvqa-------epleaaitssmtnfdilkrlnrlekevfgkqildnsd 108
Sm      LKEEVKQAKKRQEAAQTTVSPAASVSSSRVATTATNFDILKRISRLEKEVFGKQITE--- 112
Ef      lmakldqlskaqptp--rv-----aqevpksaavtnfdilkrlsnlerevfgkkldetps 109
Lm      lvqeldnaplrtstqpapt-----fqaaaqpagttnfdilkrlsnlekhvfgnklddne- 113
Bs      lkkqleeaskkq----------------pvqsnttnfdilkrlsnlekhvfgsklyd--- 98
        *  :: .                        :  ********:..**:.***.:: :

Sp      f--------------------------   109
Sm      ---------------------------   112
Ef      tpvtpsapsmtaepanhdvdnaqtrqf   136
Lm      ---------------------------   113
Bs      ---------------------------   98
```

Figure S1 ClustalO alignment of GpsB protein sequences. GpsB phosphosites in other microorganisms, highlighted in green, include Thr88 (*Listeria monocytogenes*; Lm), Thr79/Thr83 (*Streptococcus pneumoniae*; Sp), Thr75 (*Bacillus subtilis*; Bs), Ser80/Thr84 (*Enterococcus faecalis*; Ef). Predicted Streptococcus mutans (Sm) phosphosites are highlighted in yellow, Thr72, Thr73, Thr87, Thr88 (wild-type), Ser3 (Δ*pknB* and Δ*pppL*), and Ser75 (Δ*pppL*).

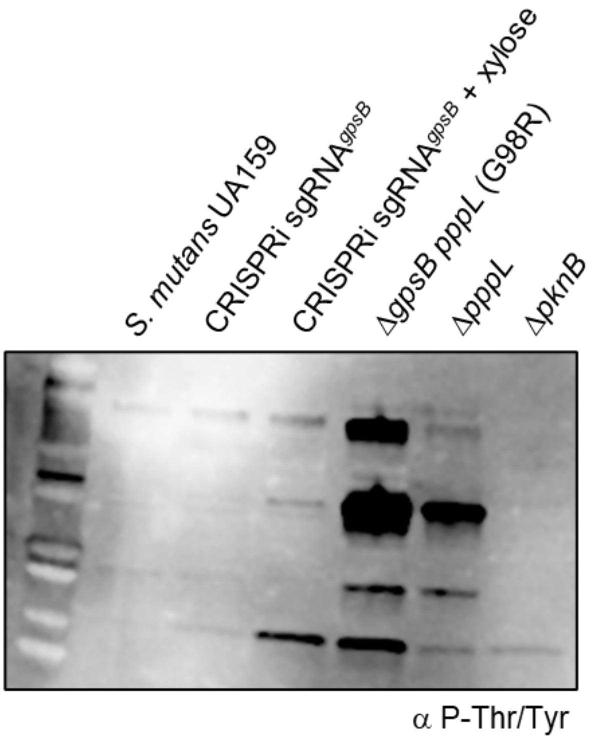

Figure S2 GpsB repression (with CRISPRi) or deletion alters threonine phosphorylation of proteins. Western blotting, with an α-pThr/Tyr antibody, was performed to detect protein phosphorylated at threonine and tyrosine residues. Cells were cultured to OD_{600} ~0.4-0.6 and collected for protein extraction. Image is representative of four biological replicates with similar results.

Table S1 Detection of phosphorylation at serine, threonine, and tyrosine residues in *S. mutans* UA159

Gene	Protein Description	Phosphorylation Site	Samples Detected*
adhE	Aldehyde-alcohol dehydrogenase	T7	3
argR	Arginine repressor	T40	5
aspB	Asparagine-oxo-acid transaminase	S242	5
aspB	Asparagine-oxo-acid transaminase	T244	5
bipA	Large ribosomal subunit assembly factor BipA	T559	2
cas1	CRISPR-associated endonuclease Cas1	Y16	5
cas1	CRISPR-associated endonuclease Cas1	S15	5
clpP	ATP-dependent Clp protease proteolytic subunit	S10	5
coaD	Phosphopantetheine adenylyltransferase	T117	5
cysE	Serine acetyltransferase	T193	3
cysK	Cysteine synthase	S120	4
divIVA	Cell division protein DivIVA	T262	12
divIVA	Cell division protein DivIVA	T77	10
divIVA	Cell division protein DivIVA	T201	5
divIVA	Cell division protein DivIVA	T232	3
divIVA	Cell division protein DivIVA	T195	3
divIVA	Cell division protein DivIVA	T4	1
efp	Elongation factor P	S184	5
efp	Elongation factor P	T145	1
eno	Enolase	T200	5
fbaA	Fructose-bisphosphate aldolase	T233	3
fbaA	Fructose-bisphosphate aldolase	Y257	1
ffh	Signal recognition particle protein	S397	15
ffh	Signal recognition particle protein	T285	1
folC	tetrahydrofolate synthase	S84	2
frr	Ribosome-recycling factor	S19	8
fruA	Fructan beta-fructosidase	S1356	15
fruA	Fructan beta-fructosidase	S1358	15

fruA	Fructan beta-fructosidase	S1355	15
fruC	Constitutive fructose permease	S114	3
fruI	Inducible fructose permease	S291	10
fruI	Inducible fructose permease	S30	7
fruI	Inducible fructose permease	S279	5
fruI	Inducible fructose permease	T57	5
fruI	Inducible fructose permease	S278	5
fruI	Inducible fructose permease	T55	3
ftsZ	Cell division protein FtsZ	S333	5
ftsZ	Cell division protein FtsZ	S4	3
fusA	Elongation factor G	S638	9
fusA	Elongation factor G	T238	5
fusA	Elongation factor G	S480	1
gapC	Glyceraldehyde-3-phosphate dehydrogenase	S212	12
glgC	Glucose-1-phosphate adenylyltransferase	S170	2
glnQ	Amino acid ABC transporter, ATP-binding protein	T247	5
glyA	Serine hydroxymethyltransferase	Y55	1
gpsB	Cell cycle protein GpsB	T72	9
gpsB	Cell cycle protein GpsB	T88	5
gpsB	Cell cycle protein GpsB	T73	4
gpsB	Cell cycle protein GpsB	T87	1
greA	Transcription elongation factor GreA	S45	5
greA	Transcription elongation factor GreA	S40	5
greA	Transcription elongation factor GreA	S48	5
groEL	Chaperonin GroEL	S460	10
groEL	Chaperonin GroEL	T355	5
groEL	Chaperonin GroEL	S356	4
groEL	Chaperonin GroEL	T385	3
groEL	Chaperonin GroEL	S349	3
groEL	Chaperonin GroEL	T268	3
groEL	Chaperonin GroEL	T25	1

groES	Co-chaperonin GroES	T20	3
groES	Co-chaperonin GroES	S30	1
gtfB	Glucosyltransferase-I	T1041	3
gtfB	Glucosyltransferase-I	S1272	1
gtfC	Glucosyltransferase-SI	T990	5
hisS	Histidine--tRNA ligase	T57	5
hup	DNA-binding protein HU	T66	5
ilvC	Ketol-acid reductoisomerase (NADP(+))	S99	4
khpB	RNA-binding protein KhpB	T88	2
ldh	L-lactate dehydrogenase	T227	4
lemA	Cytoplasmic membrane protein LemA-like protein	T170	2
lepA	Elongation factor 4	T39	10
lepA	Elongation factor 4	S41	4
lepA	Elongation factor 4	S42	2
lytS	Sensor protein LytS	T524	1
mapZ	Mid-cell-anchored protein Z	T127	15
mapZ	Mid-cell-anchored protein Z	T18	15
mapZ	Mid-cell-anchored protein Z	S22	5
mapZ	Mid-cell-anchored protein Z	T101	1
mecA	Adapter protein MecA	S57	5
mltG	Endolytic murein transglycosylase	T191	15
mltG	Endolytic murein transglycosylase	T102	15
mltG	Endolytic murein transglycosylase	T166	15
mltG	Endolytic murein transglycosylase	T105	10
mltG	Endolytic murein transglycosylase	T107	3
mltG	Endolytic murein transglycosylase	T222	2
mltG	Endolytic murein transglycosylase	T379	1
mltG	Endolytic murein transglycosylase	T374	1
mltG	Endolytic murein transglycosylase	S375	1
murD	UDP-N-acetylmuramoylalanine--D-glutamate ligase	T332	4
pepX	Xaa-Pro dipeptidyl-peptidase	S574	5

pfkA	ATP-dependent 6-phosphofructokinase	S159	7
pgi	Glucose-6-phosphate isomerase	T149	10
pknB	non-specific serine/threonine protein kinase	T291	5
polC	DNA polymerase III PolC-type	T190	1
ppc	Phosphoenolpyruvate carboxylase	S719	4
ptcC	Permease IIC component	Y437	5
ptnA	PTS system mannose-specific EIIAB component	T145	4
ptnA	PTS system mannose-specific EIIAB component	T203	2
ptnC	PTS system, mannose-specific component IIC	S261	2
ptsH	Phosphocarrier protein HPr	T12	15
ptsH	Phosphocarrier protein HPr	S27	4
pttB	PTS system, trehalose-specific IIABC component	T487	15
pttB	PTS system, trehalose-specific IIABC component	S493	15
pttB	PTS system, trehalose-specific IIABC component	S491	15
pttB	PTS system, trehalose-specific IIABC component	T485	10
pttB	PTS system, trehalose-specific IIABC component	T503	5
pttB	PTS system, trehalose-specific IIABC component	T29	4
pttB	PTS system, trehalose-specific IIABC component	S653	2
ptxB	PTS system, enzyme IIB component	S40	1
pykF	Pyruvate kinase	T412	1
pyrH	Uridylate kinase	S14	5
recA	Protein RecA	S175	5
rf2	Peptide chain release factor 2	T16	4
rgpA	RgpAc glycosyltransferase	S214	5
rnz	Ribonuclease Z	T162	5
rplI	Large ribosomal subunit protein bL9	S76	1
rplJ	Large ribosomal subunit protein uL10	S2	1
rplO	Large ribosomal subunit protein uL15	S131	10
rplQ	Large ribosomal subunit protein bL17	S74	2
rplR	Large ribosomal subunit protein uL18	S89	9
rplR	Large ribosomal subunit protein uL18	T69	1

rplW	Large ribosomal subunit protein uL23	T89	1
rpmC	Large ribosomal subunit protein uL29	T47	1
rpmF	Large ribosomal subunit protein bL32	T32	15
rpmF	Large ribosomal subunit protein bL32	S37	7
rpoA	DNA-directed RNA polymerase subunit alpha	S281	5
rpoA	DNA-directed RNA polymerase subunit alpha	S279	5
rpsE	Small ribosomal subunit protein uS5	S154	5
rpsE	Small ribosomal subunit protein uS5	S159	5
rpsH	Small ribosomal subunit protein uS8	S23	2
rpsM	Small ribosomal subunit protein uS13	S50	15
rpsO	Small ribosomal subunit protein uS15	Y53	2
rpsO	Small ribosomal subunit protein uS15	T52	2
rpsR	Small ribosomal subunit protein bS18	T31	5
rpsU	Small ribosomal subunit protein bS21	S11	2
rs1	Ribosomal protein S1 sequence specific DNA-binding protein	T198	15
rs1	Ribosomal protein S1 sequence specific DNA-binding protein	T338	4
rs1	Ribosomal protein S1 sequence specific DNA-binding protein	S123	2
scnK	histidine kinase	S413	5
scnK	histidine kinase	T257	5
sepF	Cell division protein SepF	S78	3
sepF	Cell division protein SepF	S41	1
SMU_1037c	histidine kinase	Y139	5
SMU_104	Alpha-glucosidase glycosyl hydrolase	S87	9
SMU_104	Alpha-glucosidase glycosyl hydrolase	S88	9
SMU_1080c	CwlT-like lysozyme domain-containing protein	T47	3
SMU_1143c	Riboflavin biosynthesis protein	T245	10
SMU_1157c	SIR2-like domain-containing protein	S80	1
SMU_1157c	SIR2-like domain-containing protein	S83	1
SMU_1208c	Uncharacterized protein	S158	1

SMU_1309c	Glycerol dehydrogenase	Y358	1
SMU_1367c	Methyltransferase domain-containing protein	Y28	5
SMU_160	DUF2268 domain-containing protein	Y168	5
SMU_160	DUF2268 domain-containing protein	S177	5
SMU_1621c	UPF0346 protein SMU_1621c	Y6	6
SMU_1621c	UPF0346 protein SMU_1621c	T11	6
SMU_164	tRNA/rRNA methyltransferase	T63	4
SMU_164	tRNA/rRNA methyltransferase	S65	4
SMU_1641c	CsbD-like domain-containing protein	T29	10
SMU_1681c	VOC domain-containing protein	S35	1
SMU_1772c	Uncharacterized protein	Y55	11
SMU_1772c	Uncharacterized protein	T56	5
SMU_1787c	Secreted protein	S123	10
SMU_1787c	Secreted protein	T121	5
SMU_1787c	Secreted protein	T118	5
SMU_1853	Integral membrane protein	T33	5
SMU_1876	DUF2975 domain-containing protein	S3	5
SMU_1876	DUF2975 domain-containing protein	Y4	5
SMU_1881c	ABC transporter, ATP-binding protein	S203	5
SMU_1881c	ABC transporter, ATP-binding protein	S207	5
SMU_1881c	ABC transporter, ATP-binding protein	T202	5
SMU_1896c	Uncharacterized protein	Y16	5
SMU_1956c	PTS fructose transporter subunit IA	Y11	15
SMU_1957	PTS system, mannose-specific IID component	T177	5
SMU_1958c	PTS system, mannose-specific IIC component	S269	8
SMU_1958c	PTS system, mannose-specific IIC component	S273	3
SMU_1960c	PTS system, mannose-specific IIB component	T17	15
SMU_1960c	PTS system, mannose-specific IIB component	T19	5
SMU_1961c	PTS system, sugar-specific enzyme IIA component	S135	15
SMU_1961c	PTS system, sugar-specific enzyme IIA component	T48	2
SMU_2064c	glycosyltransferase PgfM2	T16	15

SMU_2079c	UPF0297 protein SMU_2079c	T4	15
SMU_2079c	UPF0297 protein SMU_2079c	T7	15
SMU_2127	Succinate semialdehyde dehydrogenase	S214	2
SMU_333	MFS transporter	T2	4
SMU_38c	Transcriptional regulator	T41	5
SMU_393	Regulator of chromosome segregation-like C-terminal domain-containing protein	T41	5
SMU_447	UPF0291 protein SMU_447	T62	10
SMU_470	UPF0398 protein SMU_470	Y144	9
SMU_470	UPF0398 protein SMU_470	Y146	9
SMU_487	Response regulator	T131	1
SMU_501	Uncharacterized protein	S61	5
SMU_502	TcaA 4th domain-containing protein	T52	10
SMU_502	TcaA 4th domain-containing protein	S53	5
SMU_528c	ABM domain-containing protein	S83	5
SMU_530c	Integral membrane protein	S162	2
SMU_54	Amino acid recemase	T128	3
SMU_54	Amino acid recemase	Y127	3
SMU_567	Glutamine ABC transporter, permease component	S4	1
SMU_567	Glutamine ABC transporter, permease component	S7	1
SMU_632	Transcriptional regulator	T70	4
SMU_632	Transcriptional regulator	S67	4
SMU_635	Integral membrane protein	S103	10
SMU_668c	Ribonucleoside-diphosphate reductase	S157	1
SMU_695	LysM domain-containing protein	T14	5
SMU_695	LysM domain-containing protein	T15	5
SMU_720	Probable membrane transporter protein	T157	4
SMU_739c	Adhesin	T472	4
SMU_752	Protein SprT-like	T4	5
SMU_752	Protein SprT-like	Y6	5
SMU_757	Gas vesicle protein	T76	10

SMU_768c	YolD-like protein	S54	1
SMU_874	Hcy-binding domain-containing protein	S480	5
SMU_874	Hcy-binding domain-containing protein	T482	5
SMU_913	Glutamate dehydrogenase	T192	5
sodA	Superoxide dismutase [Mn/Fe]	T123	4
sodA	Superoxide dismutase [Mn/Fe]	T202	3
ssb	Single-stranded DNA-binding protein	T12	5
thrC	Threonine synthase	T2	4
tig	Trigger factor	T40	5
tig	Trigger factor	S385	2
tig	Trigger factor	S400	2
treA	Alpha,alpha-phosphotrehalase	S391	5
tsf	Elongation factor Ts	T5	7
tuf	Elongation factor Tu	S52	15
tuf	Elongation factor Tu	T387	10
tuf	Elongation factor Tu	T261	5
tuf	Elongation factor Tu	Y50	3
tuf	Elongation factor Tu	S390	3
valS	Valine--tRNA ligase	S6	5
whiA	Probable cell division protein WhiA	T225	1

*phosphosites were identified from a total of 15 biological replicates

Table S2 Proteomics comparison of *S. mutans* UA159 vs *S. mutans* Δ*pknB*

Gene	Description	log2FC	adj.P.Val
SMU_209c	DUF961 domain-containing protein	4.75	9.26E-04
SMU_205c	Bacteriocin immunity protein	4.71	8.02E-04
SMU_210c	Uncharacterized protein	3.98	1.15E-03
SMU_1486c	Histidinol-phosphatase	3.90	2.01E-07
SMU_201c	Transposon protein	3.77	4.36E-03
SMU_753	Phage shock protein PspC N-terminal domain-containing protein	3.70	2.43E-07
bglA	Beta-glucosidase	3.14	2.45E-08
SMU_214c	Uncharacterized protein	3.04	6.51E-04
SMU_752	Protein SprT-like	2.89	3.62E-07
SMU_195c	Phage protein	2.85	1.03E-02
SMU_505	Methyltransferase	2.80	4.73E-07
SMU_503c	Lipoprotein	2.66	6.03E-06
SMU_758c	DUF3270 family protein	2.26	9.79E-05
SMU_1402c	Uncharacterized protein	2.26	6.07E-09
pepV	Dipeptidase	2.25	6.13E-03
SMU_2084c	Uncharacterized protein	2.22	3.38E-09
cas2	CRISPR-associated endoribonuclease Cas2	2.20	4.58E-10
lguL	Aldoketomutase	2.15	4.99E-03
cas9	CRISPR-associated endonuclease Cas9	2.14	4.38E-08
gatC	Aspartyl/glutamyl-tRNA(Asn/Gln) amidotransferase subunit C	2.00	5.61E-06
SMU_820	HlyD family secretion protein	2.00	3.52E-06
SMU_348	Histidine triad (HIT) hydrolase	1.90	3.16E-08
SMU_211c	Integral membrane protein	1.89	1.77E-04
SMU_955	Integral membrane protein	1.84	3.49E-05
eno	Enolase	1.83	1.42E-03
SMU_802	Extracellular protein	1.77	4.97E-05

SMU_984	Peptidase C51 domain-containing protein	1.76	1.78E-09
SMU_1067c	ABC transporter, permease protein	1.70	1.47E-06
malF	Maltose/maltodextrin transport system permease protein	1.68	1.13E-06
SMU_1125c	Methyltransferase small domain-containing protein	1.66	2.85E-06
nrdD	Anaerobic ribonucleoside-triphosphate reductase	1.65	4.94E-07
SMU_893	Anticodon nuclease	1.62	3.66E-03
acn	Aconitate hydratase A	1.61	3.63E-05
SMU_737	Serine aminopeptidase S33 domain-containing protein	1.60	5.34E-06
SMU_1070c	HTH LytTR-type domain-containing protein	1.59	4.64E-07
SMU_1044c	Pseudouridine synthase	1.57	3.58E-06
SMU_1087	Probable tautomerase SMU_1087	1.54	4.52E-03
SMU_1119c	Sugar ABC transporter, permease protein	1.54	1.25E-07
SMU_1790c	Transcriptional regulator	1.53	5.77E-06
SMU_797	DUF4649 domain-containing protein	1.51	1.29E-05
malQ	4-alpha-glucanotransferase	1.46	5.27E-07
malX	Maltose/maltodextrin ABC transporter, sugar-binding protein MalX	1.46	1.07E-08
SMU_1068c	ABC transporter, ATP-binding protein	1.46	1.53E-07
SMU_630	Beta-lactamase class A catalytic domain-containing protein	1.45	8.65E-08
SMU_2134	Transcriptional regulator	1.42	4.67E-08
SMU_1616c	Nudix hydrolase domain-containing protein	1.39	4.42E-08
SMU_217c	Uncharacterized protein	1.39	6.26E-04
SMU_1118c	ABC sugar transporter, permease protein	1.35	5.97E-06
rpoZ	DNA-directed RNA polymerase subunit omega	1.33	2.54E-05
SMU_1576c	AAA+ ATPase domain-containing protein	1.32	1.42E-08
phoH	PhoH-like protein	1.30	3.73E-06
SMU_1069c	DUF3021 domain-containing protein	1.29	5.55E-08

nrdG	Ribonucleoside-diphosphate reductase subunit beta	1.28	1.12E-05
adhE	Aldehyde-alcohol dehydrogenase	1.27	4.28E-04
SMU_668c	Ribonucleoside-diphosphate reductase	1.26	1.17E-06
metE	5-methyltetrahydropteroyltriglutamate--homocysteine methyltransferase	1.24	3.05E-08
rnhB	Ribonuclease HII	1.21	5.55E-07
SMU_197c	Uncharacterized protein	1.21	5.32E-03
SMU_1571	ABC transporter, ATP-binding protein, MsmK-like protein	1.20	9.33E-07
SMU_1083c	L-threonylcarbamoyladenylate synthase	1.19	9.33E-08
SMU_933	Amino acid ABC transporter, periplasmic amino acid-binding protein	1.19	7.67E-07
adcA	Surface adhesin AdcA protein-like protein putative Zn-binding lipoprotein	1.19	2.57E-06
rnz	Ribonuclease Z	1.18	1.45E-06
SMU_1256c	Phage protein	1.18	2.72E-06
SMU_1609c	Protein-export membrane protein SecG	1.17	7.45E-04
SMU_936	Amino acid ABC transporter, ATP-binding protein	1.16	9.29E-08
SMU_1375c	Uncharacterized protein	1.16	9.20E-06
recO	DNA repair protein RecO	1.16	5.27E-06
apbE	FAD:protein FMN transferase	1.16	7.85E-07
coaC	Probable phosphopantothenoylcysteine decarboxylase	1.16	3.64E-06
metB	Cystathionine gamma-synthase possible bifunctional enzyme	1.15	5.32E-06
SMU_1245c	Hydrolase	1.15	1.72E-07
SMU_1081c	Pseudouridylate synthases, 23S RNA-specific	1.14	3.89E-08
xseB	Exodeoxyribonuclease 7 small subunit	1.14	1.06E-04
SMU_930c	Transcriptional regulator	1.14	2.48E-06
SMU_1279c	Cell division protein (Cell shape determining protein)	1.14	3.33E-06

SMU_411c	Methyl-accepting chemotaxis protein	1.10	5.80E-06
SMU_1120	Sugar ABC transporter, ATP-binding protein	1.10	3.37E-07
icd	Isocitrate dehydrogenase [NADP]	1.10	1.42E-04
SMU_1464c	SAM-dependent methyltransferase	1.09	3.88E-07
SMU_1303c	Dipeptidase	1.09	2.27E-05
SMU_1412c	ABC transporter, membrane protein subunit and ATP-binding protein	1.08	8.45E-05
SMU_587	SGNH hydrolase-type esterase domain-containing protein	1.07	2.65E-05
SMU_906	ABC transporter, ATP-binding protein	1.07	2.18E-07
SMU_1577c	YobI-like P-loop NTPase domain-containing protein	1.06	3.40E-08
pncA	Pyrazinamidase/nicotinamidase	1.05	7.58E-02
potA	Spermidine/putrescine import ATP-binding protein PotA	1.05	9.35E-08
polC	DNA polymerase III PolC-type	1.05	1.73E-06
SMU_857	Uracil permease	1.04	1.17E-05
cysD	O-acetylhomoserine sulfhydrylase	1.04	5.78E-04
argG	Argininosuccinate synthase	1.04	2.57E-06
SMU_198c	Conjugative transposon protein	1.04	2.28E-05
phsG	Alpha-1,4 glucan phosphorylase	1.03	2.35E-05
SMU_1168	Transcriptional regulator	1.03	4.28E-05
SMU_962	Dehydrogenase	1.03	1.70E-04
SMU_756	General stress protein	1.02	1.30E-04
cas1	CRISPR-associated endonuclease Cas1	1.01	8.80E-07
SMU_804	FRG domain-containing protein	1.01	4.95E-07
SMU_635	Integral membrane protein	1.01	1.22E-06
gpmA	2,3-bisphosphoglycerate-dependent phosphoglycerate mutase	1.01	2.85E-04
SMU_1647c	Transcriptional regulator	-1.00	2.20E-06
psaB	ABC transporter, permease protein	-1.00	5.16E-06
SMU_100	Sorbose PTS system, IIB component	-1.01	3.44E-06

SMU_409	tRNA threonylcarbamoyladenosine biosynthesis protein TsaE	-1.01	1.95E-08
rnpA	Ribonuclease P protein component	-1.01	5.87E-07
rplI	Large ribosomal subunit protein bL9	-1.01	5.27E-08
hpf	Ribosome hibernation promoting factor	-1.02	4.73E-05
mtlA	PTS system mannitol-specific EIICB component	-1.03	6.98E-06
dexA	Dextranase	-1.04	1.33E-03
gtfD	Glucosyltransferase-S	-1.06	8.71E-06
SMU_1112c	VOC domain-containing protein	-1.06	2.52E-07
SMU_1960c	PTS system, mannose-specific IIB component	-1.08	1.82E-03
rgpC	Transport permease protein	-1.10	1.20E-04
SMU_2073c	30S ribosomal protein S10	-1.11	3.52E-07
purD	Phosphoribosylamine--glycine ligase	-1.13	2.83E-07
trpD	Anthranilate phosphoribosyltransferase	-1.13	4.65E-07
adk	Adenylate kinase	-1.15	7.85E-06
copZ	Copper chaperone	-1.16	5.59E-04
SMU_1278c	Hydrolase	-1.17	2.81E-07
trpF	N-(5-phosphoribosyl)anthranilate isomerase	-1.18	1.58E-07
SMU_1904c	Bacteriocin transport accessory protein	-1.19	2.37E-04
SMU_1442c	DUF1307 domain-containing protein	-1.19	5.38E-06
sodA	Superoxide dismutase [Mn/Fe]	-1.20	1.28E-06
SMU_1615c	DinB-like domain-containing protein	-1.21	3.72E-04
hisC	Histidinol-phosphate aminotransferase	-1.21	2.06E-08
SMU_1434c	Glycosyltransferase	-1.21	7.27E-06
tpx	Thiol peroxidase	-1.24	4.08E-06
SMU_1927	ABC transporter, ATP-binding protein	-1.25	8.91E-03
SMU_1213c	5-nucleotidase	-1.26	8.01E-04
SMU_1237c	SnoaL-like domain-containing protein	-1.27	3.67E-08
SMU_1641c	CsbD-like domain-containing protein	-1.27	4.10E-05
SMU_992	Uncharacterized protein	-1.29	8.65E-08

SMU_1976c	Uncharacterized protein	-1.32	2.76E-06
cah	Carbonic anhydrase	-1.34	5.88E-05
SMU_1723c	HD domain-containing protein	-1.36	1.22E-05
SMU_2057c	Cadmium-transporting ATPase P-type ATPase	-1.38	1.52E-03
SMU_1152c	DUF1858 domain-containing protein	-1.41	1.29E-07
bacD	Bacitracin synthetase	-1.42	1.01E-07
recG	ATP-dependent DNA helicase RecG	-1.42	2.45E-06
SMU_508	Hydrolase	-1.43	1.25E-07
SMU_1966c	Periplasmic sugar-binding protein	-1.44	3.06E-06
SMU_1436c	Uncharacterized protein	-1.44	2.20E-06
SMU_298	CoA-binding domain-containing protein	-1.44	1.72E-07
rpmB	Large ribosomal subunit protein bL28	-1.45	2.53E-08
hisD	Histidinol dehydrogenase	-1.46	5.55E-08
SMU_531	Chorismate mutase	-1.50	9.62E-05
SMU_1961c	PTS system, sugar-specific enzyme IIA component	-1.51	2.82E-04
epsC	UDP-N-acetylglucosamine 2-epimerase (non-hydrolyzing)	-1.53	1.11E-08
bacT	Thioesterase BacT	-1.55	4.92E-07
SMU_1343c	Polyketide synthase	-1.55	1.21E-07
trxA	Thioredoxin	-1.58	3.31E-06
trpC	Indole-3-glycerol phosphate synthase	-1.64	4.42E-08
hisF	Imidazole glycerol phosphate synthase subunit HisF	-1.64	3.76E-08
SMU_61	Transcriptional regulator	-1.65	7.19E-08
SMU_1788c	Bacterocin transport accessory protein, Bta	-1.66	1.12E-06
hisH	Imidazole glycerol phosphate synthase subunit HisH	-1.67	3.21E-09
lacG	6-phospho-beta-galactosidase	-1.68	5.85E-05
SMU_448	PepSY domain-containing protein	-1.70	6.41E-10
ptxA	Ascorbate-specific PTS system EIIA component	-1.72	8.65E-08
lacE	PTS system lactose-specific EIICB component	-1.74	2.28E-03

fruA	Fructan beta-fructosidase	-1.78	2.52E-07
SMU_1348c	ABC transporter ATP-binding protein	-1.78	6.41E-10
SMU_1548c	histidine kinase	-1.80	1.96E-05
yjdM	Protein YjdM	-1.82	3.55E-09
bgl	Phospho-beta-glucosidase	-1.84	3.67E-08
SMU_1347c	ABC3 transporter permease C-terminal domain-containing protein	-1.87	1.53E-07
SMU_1344c	[acyl-carrier-protein] S-malonyltransferase	-1.92	3.72E-08
hisA	1-(5-phosphoribosyl)-5-[(5-phosphoribosylamino)methylideneamino] imidazole-4-carboxamide isomerase	-1.93	1.95E-08
serB	phosphoserine phosphatase	-2.00	7.73E-09
gbpA	Glucan-binding protein A, GbpA	-2.01	4.91E-06
lacC	Tagatose-6-phosphate kinase	-2.03	2.74E-04
lacD1	Tagatose 1,6-diphosphate aldolase 1	-2.14	2.27E-04
ftf	Levansucrase	-2.24	1.49E-06
SMU_609	40K cell wall protein	-2.29	3.17E-08
bacA2	Surfactin synthetase	-2.34	7.66E-09
SMU_1345c	Peptide synthetase	-2.42	1.54E-09
gbpC	Glucan-binding protein C, GbpC	-2.46	2.60E-06
bacA1	Bacitracin synthetase 1 BacA	-2.52	1.07E-08
lacD2	Tagatose 1,6-diphosphate aldolase 2	-2.53	2.48E-07
rpmD	Large ribosomal subunit protein uL30	-2.57	2.42E-10
SMU_1341c	Gramicidin S synthetase	-2.59	2.80E-09
lacB	Galactose-6-phosphate isomerase subunit LacB	-2.59	3.94E-05
SMU_843	Capsule synthesis protein CapA domain-containing protein	-2.60	8.84E-07
SMU_63c	Carbohydrate-binding domain-containing protein	-2.63	3.33E-07
SMU_311	PTS system, sorbitol (Glucitol) phosphotransferase enzyme IIC2	-2.75	4.58E-10
lacA	Galactose-6-phosphate isomerase subunit LacA	-2.76	8.67E-05

ptxB	PTS system, enzyme IIB component	-2.84	1.58E-07
gbpB	Secreted antigen GbpB/SagA putative peptidoglycan hydrolase	-2.90	3.67E-08
wapE	Gram-positive cocci surface proteins LPxTG domain-containing protein	-2.92	1.30E-08
SMU_367	Putative hydrolase SMU_367	-2.93	7.66E-09
SMU_05	DUF951 domain-containing protein	-2.97	5.01E-07
ptsH	Phosphocarrier protein HPr	-2.99	7.66E-09
fruC	Constitutive fructose permease	-3.09	1.00E-07
SMU_313	PTS system, sorbitol-specific enzyme IIA	-3.10	1.03E-09
ssb2	Single-stranded DNA-binding protein	-3.11	3.17E-08
pdhC	Dihydrolipoamide acetyltransferase component of pyruvate dehydrogenase complex	-3.16	6.38E-11
lacF	PTS system lactose-specific EIIA component	-3.17	4.96E-05
fruD	Constitutive fructose permease	-3.28	4.99E-07
pdhA	Pyruvate dehydrogenase, TPP-dependent E1 component alpha-subunit	-3.37	3.01E-11
SMU_571	FeoB-associated Cys-rich membrane protein	-3.44	1.92E-04
SMU_308	Sorbitol-6-phosphate 2-dehydrogenase	-3.49	9.76E-08
SMU_626	Competence protein	-3.50	6.31E-07
ptcB	PTS system, cellobiose-specific IIB component	-3.51	5.91E-07
SMU_689	Lysozyme	-3.64	1.38E-07
gtfC	Glucosyltransferase-SI	-3.71	6.62E-10
SMU_312	PTS system, sorbitol phosphotransferase enzyme IIBC	-3.88	4.58E-10
rpmJ	Large ribosomal subunit protein bL36	-3.89	4.21E-09
pdhB	Pyruvate dehydrogenase E1 component beta subunit	-4.27	1.14E-10
gtfB	Glucosyltransferase-I	-4.34	1.56E-07
wapA	Wall-associated protein	-4.56	3.55E-09
pknB	non-specific serine/threonine protein kinase	-4.73	5.66E-11
SMU_2147c	LysM domain-containing protein	-5.01	6.21E-10

SMU_575c	Membrane protein	-7.20	5.66E-11

Table S3 Proteomics comparison of *S. mutans* UA159 vs *S. mutans* Δ*pppL*

Gene	Description	log2FC	adj.P.Val
SMU_1904c	Bacteriocin transport accessory protein	6.38	9.17E-07
SMU_1013c	Mg2+/citrate transporter	4.92	4.71E-03
SMU_195c	Phage protein	4.56	1.45E-02
SMU_1909c	Uncharacterized protein	4.49	1.59E-06
SMU_1832	Uncharacterized protein	3.83	5.11E-03
oadB	Oxaloacetate decarboxylase, sodium ion pump subunit	3.48	3.57E-02
ftf	Levansucrase	3.37	9.71E-04
SMU_205c	Bacteriocin immunity protein	3.03	2.81E-02
SMU_690	DUF6287 domain-containing protein	2.93	9.64E-03
cilB	Citrate lyase subunit beta	2.93	1.37E-02
pycB	Pyruvate carboxylase/oxaloacetate decarboxylase, alpha subunit	2.86	2.02E-02
SMU_1067c	ABC transporter, permease protein	2.75	9.20E-03
ptcB	PTS system, cellobiose-specific IIB component	2.71	9.85E-02
SMU_1069c	DUF3021 domain-containing protein	2.69	4.37E-03
SMU_1012c	Transcriptional regulator	2.68	1.08E-02
pdhC	Dihydrolipoamide acetyltransferase component of pyruvate dehydrogenase complex	2.60	1.67E-01
SMU_1014	HEAT repeat domain-containing protein	2.58	1.08E-02
SMU_609	40K cell wall protein	2.57	8.70E-02
citD	Citrate lyase acyl carrier protein	2.42	4.61E-03
SMU_2134	Transcriptional regulator	2.41	1.89E-06
msmG	Multiple sugar-binding transport system permease protein MsmG	2.40	1.74E-02
SMU_1068c	ABC transporter, ATP-binding protein	2.33	5.49E-03
pdhD	Dihydrolipoyl dehydrogenase	2.28	3.47E-01
SMU_753	Phage shock protein PspC N-terminal domain-containing protein	2.28	1.61E-03

fruC	Constitutive fructose permease	2.20	1.74E-01
pdhA	Pyruvate dehydrogenase, TPP-dependent E1 component alpha-subunit	2.18	2.48E-01
gtfB	Glucosyltransferase-I	2.15	2.81E-02
bcc	Biotin carboxyl carrier protein	2.15	2.55E-02
SMU_1946	DUF4956 domain-containing protein	2.08	9.90E-03
trkH	Potassium uptake protein TrkH	2.07	8.67E-03
SMU_201c	Transposon protein	2.06	3.85E-02
SMU_1685c	DUF1803 domain-containing protein	2.03	1.68E-02
glgB	1,4-alpha-glucan branching enzyme GlgB	2.03	1.27E-01
SMU_527	Uncharacterized protein	1.99	6.85E-02
SMU_1722c	Integral membrane protein	1.98	8.58E-03
SMU_331	Transcriptional regulator	1.97	6.77E-03
SMU_840c	Histone acetyltransferase Gcn5	1.97	9.13E-03
SMU_2079c	UPF0297 protein SMU_2079c	1.97	1.32E-03
pdhB	Pyruvate dehydrogenase E1 component beta subunit	1.96	1.25E-01
SMU_174c	Polymerase nucleotidyl transferase domain-containing protein	1.96	2.45E-03
satE	DUF3307 domain-containing protein	1.94	8.35E-03
SMU_329	HTH DNA-binding protein	1.92	9.60E-03
clpB	Chaperone protein ClpB	1.91	3.09E-02
xseB	Exodeoxyribonuclease 7 small subunit	1.91	7.75E-03
SMU_1454c	Membrane protein possible permease	1.91	1.27E-02
SMU_627	DUF805 domain-containing protein	1.89	5.23E-02
SMU_1479	DUF3042 domain-containing protein	1.85	2.87E-02
SMU_133c	MDR permease transmembrane efflux protein	1.85	1.76E-02
SMU_210c	Uncharacterized protein	1.84	3.35E-02
dexA	Dextranase	1.83	6.93E-04
SMU_626	Competence protein	1.83	4.16E-02

citC	[Citrate [pro-3S]-lyase] ligase	1.81	1.25E-02
msmF	Multiple sugar-binding transport system permease protein MsmF	1.80	2.38E-02
SMU_198c	Conjugative transposon protein	1.80	1.26E-02
opuCd	Osmoprotectant ABC transporter permease protein	1.80	4.87E-02
SMU_1279c	Cell division protein (Cell shape determining protein)	1.79	6.77E-03
SMU_1609c	Protein-export membrane protein SecG	1.75	9.71E-03
SMU_602	Sodium-dependent transporter	1.73	8.00E-03
secE	Preprotein translocase subunit SecE	1.73	2.43E-02
adhE	Aldehyde-alcohol dehydrogenase	1.73	2.60E-02
atpB	ATP synthase subunit a	1.73	1.23E-02
satD	DNA-binding protein	1.71	1.57E-02
SMU_273	Hexulose-6-phosphate synthase	1.71	5.29E-03
fruD	Constitutive fructose permease	1.71	5.68E-02
SMU_1488c	DUF3884 family protein	1.71	2.47E-02
SMU_1694c	Permease	1.70	1.68E-02
SMU_274	L-ribulose-5-phosphate 3-epimerase	1.69	5.58E-03
sgaT	Ascorbate-specific PTS system EIIC component	1.67	2.53E-02
opuCb	Osmoprotectant ABC transporter permease protein	1.67	4.68E-02
dnaE	DNA polymerase III subunit alpha	1.67	1.90E-02
bgl	Phospho-beta-glucosidase	1.66	5.66E-02
SMU_405c	Transcriptional regulator	1.65	1.08E-02
SMU_661	Transcriptional regulator	1.65	4.37E-03
SMU_89c	Nitrite transporter	1.65	4.32E-02
SMU_209c	DUF961 domain-containing protein	1.64	2.57E-02
SMU_756	General stress protein	1.63	1.08E-02
SMU_2127	Succinate semialdehyde dehydrogenase	1.63	1.53E-02
SMU_802	Extracellular protein	1.61	7.56E-03

ecfT	Energy-coupling factor transporter transmembrane protein EcfT	1.59	1.89E-02
SMU_1703c	Riboflavin transporter	1.59	1.09E-02
ftsW	Probable peptidoglycan glycosyltransferase FtsW	1.59	1.21E-02
SMU_1704	Transcription regulator PadR N-terminal domain-containing protein	1.59	4.37E-03
SMU_1725	acylphosphatase	1.59	6.89E-03
SMU_2161c	Permease	1.58	1.52E-02
aguA	Putative agmatine deiminase	1.58	3.83E-02
SMU_1555c	Uncharacterized protein	1.57	1.53E-02
rpsT	Small ribosomal subunit protein bS20	1.57	1.37E-02
SMU_414	ABC transporter, permease protein	1.57	1.82E-02
ptcA	Putrescine carbamoyltransferase	1.57	1.05E-01
SMU_1765c	WYL domain-containing protein	1.57	1.82E-02
SMU_235	Band 7 domain-containing protein	1.57	1.33E-02
clpP	ATP-dependent Clp protease proteolytic subunit	1.56	2.31E-02
SMU_1213c	5-nucleotidase	1.54	8.00E-03
SMU_275	L-ribulose-5-phosphate 4-epimerase	1.54	5.08E-03
rgpC	Transport permease protein	1.54	5.75E-02
lguL	Aldoketomutase	1.52	7.17E-02
pncA	Pyrazinamidase/nicotinamidase	1.52	1.17E-02
cilA	Citrate lyase alpha chain	1.52	9.80E-03
SMU_305	Uncharacterized protein	1.52	8.35E-03
SMU_1935c	UPF0397 protein SMU_1935c	1.51	2.39E-02
SMU_643	Esterase	1.50	1.00E-02
trkB	Trk system potassium uptake protein TrkA	1.50	1.57E-02
SMU_833	Glycosyltransferase	1.50	2.02E-02
SMU_999	Uncharacterized protein	1.50	1.18E-02
SMU_101	Sorbose PTS system, IIC component	1.49	1.31E-02

SMU_63c	Carbohydrate-binding domain-containing protein	1.49	2.61E-02
SMU_1681c	VOC domain-containing protein	1.49	4.71E-03
SMU_404c	DUF3899 domain-containing protein	1.49	1.33E-02
SMU_510c	Lipoprotein	1.47	2.81E-02
SMU_1446c	ABC transporter, permease protein	1.46	1.66E-02
ptcA	PTS system, cellobiose-specific IIA component	1.46	9.35E-02
trk	Trk system potassium uptake protein TrkA	1.45	1.20E-02
SMU_1292c	TVP38/TMEM64 family membrane protein	1.45	9.90E-03
SMU_757	Gas vesicle protein	1.45	3.52E-03
adcC	ABC transporter, ATP-binding protein	1.45	2.13E-02
rpe	Ribulose-phosphate 3-epimerase	1.45	1.06E-02
acpS	Holo-[acyl-carrier-protein] synthase	1.44	1.41E-02
SMU_651c	ABC transporter, substrate-binding protein	1.44	1.37E-02
SMU_102	PTS system, IID component	1.44	1.68E-02
psaB	ABC transporter, permease protein	1.42	1.23E-02
SMU_834	Glycosyltransferase 2-like domain-containing protein	1.41	1.68E-02
SMU_662	Protease	1.41	2.11E-02
glnP	Amino acid ABC transporter, integral membrane protein	1.40	1.56E-02
SMU_1087	Probable tautomerase SMU_1087	1.39	1.92E-02
SMU_132	Hippurate amidohydrolase	1.39	1.46E-02
SMU_720	Probable membrane transporter protein	1.38	1.21E-02
pepV	Dipeptidase	1.38	7.95E-02
cdsA	Phosphatidate cytidylyltransferase	1.38	1.90E-02
livH	Branched chain amino acid ABC transporter, permease protein	1.37	1.88E-02
SMU_940c	Hemolysin III	1.37	1.02E-01
malF	Maltose/maltodextrin transport system permease protein	1.37	2.33E-02
SMU_1852	Magnesium/cobalt transport protein	1.37	2.34E-02

lemA	Cytoplasmic membrane protein LemA-like protein	1.37	5.06E-02
SMU_857	Uracil permease	1.36	2.33E-02
SMU_1007	ABC transporter, permease protein	1.36	1.93E-02
SMU_807	Membrane protein	1.36	1.68E-02
SMU_384	HTH marR-type domain-containing protein	1.35	2.32E-02
SMU_1445c	ABC transporter, ATP-binding protein	1.34	2.38E-02
SMU_799c	Fluoroacetyl-CoA-specific thioesterase-like domain-containing protein	1.33	5.35E-02
SMU_832	Serotype determinant, transmembrane protein	1.33	3.53E-02
SMU_618	Uncharacterized protein	1.32	2.73E-02
ecfA1	Energy-coupling factor transporter ATP-binding protein EcfA1	1.32	2.10E-02
SMU_806c	Glutamine ABC transporter, permease protein	1.31	7.61E-03
SMU_1784c	Eep protein-like protein	1.31	1.65E-02
pacL	Cation-transporting P-type ATPase PacL	1.31	7.88E-03
SMU_1546	GtrA/DPMS transmembrane domain-containing protein	1.30	1.08E-02
SMU_303	Metallo-beta-lactamase domain-containing protein	1.30	4.71E-03
SMU_1641c	CsbD-like domain-containing protein	1.29	1.09E-02
SMU_695	LysM domain-containing protein	1.29	8.00E-03
SMU_1879	PTS system, mannose-specific component IID	1.29	5.70E-02
folT	Folate transporter FolT	1.29	3.12E-02
SMU_835	Glucosyltransferase	1.28	1.40E-02
rgpG	Glycosyl transferase N-acetylglucosaminyltransferase, RgpG	1.28	1.45E-02
pgsA	CDP-diacylglycerol--glycerol-3-phosphate 3-phosphatidyltransferase	1.28	1.68E-02
SMU_1443c	Tributyrin esterase	1.27	9.31E-03
SMU_460	Amino acid ABC transporter, permease	1.27	3.75E-02
SMU_413	ABC transporter, ATP-binding protein	1.27	1.88E-02
SMU_935	Amino acid ABC transporter, permease protein	1.27	1.21E-02

opuAb	ABC transporter, proline/glycine betaine permease protein	1.26	3.83E-02
SMU_1593c	CDP-diglyceride synthetase	1.26	2.06E-02
SMU_1876	DUF2975 domain-containing protein	1.26	5.96E-02
SMU_843	Capsule synthesis protein CapA domain-containing protein	1.26	1.68E-02
mntH	Divalent metal cation transporter MntH	1.26	3.18E-02
SMU_1450	Amino acid permease	1.25	2.42E-02
SMU_10	Cell division protein DivIC	1.25	2.29E-02
SMU_1719c	UPF0154 protein SMU_1719c	1.24	2.03E-02
oppC	Transmembrane protein, permease OppC	1.24	2.06E-02
atpH	ATP synthase subunit delta	1.24	6.95E-03
SMU_1660c	Initiation-control protein YabA	1.24	2.45E-03
SMU_911c	Acid-resistance membrane protein	1.24	1.27E-02
ftsL	Cell division protein FtsL	1.24	8.35E-03
SMU_2058	Transcriptional regulator	1.23	1.46E-02
ecfA2	Energy-coupling factor transporter ATP-binding protein EcfA2	1.23	2.48E-02
SMU_1164c	ABC transporter, ATP-binding protein	1.23	1.24E-02
SMU_1309c	Glycerol dehydrogenase	1.23	6.93E-04
SMU_1119c	Sugar ABC transporter, permease protein	1.22	6.45E-03
folA	7,8-dihydroneopterin aldolase	1.22	8.90E-03
SMU_167	Uncharacterized protein	1.22	1.64E-02
SMU_242c	Amino acid ABC transporter, permease protein, glutamine transport system	1.22	1.95E-02
ptnC	PTS system, mannose-specific component IIC	1.21	5.71E-02
SMU_1827	Biotin transporter	1.21	1.42E-02
SMU_354	DNA recombination protein RmuC	1.21	3.15E-02
SMU_1118c	ABC sugar transporter, permease protein	1.20	1.51E-02
mleS	Malolactic enzyme	1.20	1.61E-03

gbpC	Glucan-binding protein C, GbpC	1.20	1.04E-03
SMU_290	Metallo-beta-lactamase domain-containing protein	1.20	5.35E-02
SMU_1938c	ABC transporter, permease protein	1.20	5.94E-02
msmE	Multiple sugar-binding protein	1.19	3.71E-02
atpC	ATP synthase epsilon chain	1.19	1.68E-02
glpF	Glycerol uptake facilitator protein	1.19	6.70E-02
glgC	Glucose-1-phosphate adenylyltransferase	1.19	1.94E-01
SMU_145	Major facilitator superfamily (MFS) profile domain-containing protein	1.19	3.32E-02
atpF	ATP synthase subunit b	1.19	1.17E-02
pepB	Oligopeptidase	1.18	4.37E-03
lgt	Phosphatidylglycerol--prolipoprotein diacylglyceryl transferase	1.18	2.02E-02
ccdA	Cytochrome C biogenesis protein	1.17	1.68E-02
SMU_673	ABC transporter permease	1.17	9.26E-03
SMU_1616c	Nudix hydrolase domain-containing protein	1.17	5.44E-02
lacD2	Tagatose 1,6-diphosphate aldolase 2	1.17	3.07E-02
SMU_411c	Methyl-accepting chemotaxis protein	1.17	2.45E-03
ptxB	PTS system, enzyme IIB component	1.17	5.75E-02
gldA	Glycerol dehydrogenase	1.17	6.90E-02
fruP	Tagatose-6-phosphate kinase	1.16	6.83E-03
SMU_1470c	GyrI-like small molecule binding domain-containing protein	1.16	4.37E-03
SMU_1676c	Membrane protein	1.16	1.91E-02
pstC1	Phosphate transport system permease protein	1.16	1.28E-02
SMU_1179c	Amino acid ABC transporter, permease protein	1.16	2.95E-02
SMU_1995c	Transcriptional regulator	1.16	1.42E-02
mraY	Phospho-N-acetylmuramoyl-pentapeptide-transferase	1.15	2.91E-02
SMU_728	Oxidoreductase	1.14	8.18E-03

SMU_1800c	CRM domain-containing protein	1.14	1.45E-03
SMU_1545c	Citrulline cluster-linked protein	1.13	1.72E-02
SMU_1936c	DNA-directed RNA polymerase subunit delta	1.12	1.74E-02
SMU_118c	S-formylglutathione hydrolase	1.12	2.73E-03
SMU_1100c	Permease	1.12	1.42E-02
SMU_16	Amino acid permease	1.12	4.58E-02
SMU_1787c	Secreted protein	1.11	1.57E-02
SMU_134	Transcriptional regulator (TetR/AcrR family)	1.11	1.77E-02
SMU_501	Uncharacterized protein	1.11	2.06E-02
glgD	Glycogen biosynthesis protein GlgD	1.11	1.72E-01
plsY	Glycerol-3-phosphate acyltransferase	1.10	2.86E-02
SMU_1165c	Transcription regulator	1.09	6.73E-03
SMU_179	NADPH-dependent FMN reductase-like domain-containing protein	1.09	7.79E-02
SMU_1152c	DUF1858 domain-containing protein	1.09	8.00E-03
SMU_139	Cupin type-1 domain-containing protein	1.09	1.50E-03
atpA	ATP synthase subunit alpha	1.08	3.79E-03
oppB	Oligopeptide transport system, permease protein OppB	1.08	2.06E-02
SMU_739c	Adhesin	1.08	1.93E-01
glgA	Glycogen synthase	1.08	5.33E-02
SMU_218	Transcriptional regulator	1.08	3.26E-03
SMU_1611c	Permease possible multi-drug resistance efflux pump	1.08	4.02E-02
comEB	Deoxycytidylate deaminase	1.08	1.02E-02
gatC	Aspartyl/glutamyl-tRNA(Asn/Gln) amidotransferase subunit C	1.07	3.34E-02
SMU_1219c	MmcQ-like protein	1.07	4.37E-03
SMU_929c	Integral membrane protein	1.07	1.09E-02
SMU_05	DUF951 domain-containing protein	1.07	2.16E-01
grk	Glycerate kinase	1.06	1.45E-03

SMU_1705	DUF1700 domain-containing protein	1.05	3.53E-02
lepB	Signal peptidase I	1.05	2.97E-02
SMU_632	Transcriptional regulator	1.05	1.02E-02
potD	ABC transporter, periplasmic spermidine/putrescine-binding protein	1.05	2.73E-02
atpD	ATP synthase subunit beta	1.05	3.58E-03
rpsU	Small ribosomal subunit protein bS21	1.04	4.86E-02
opuCc	ABC transporter osmoprotectant-binding protein, glycine betaine/carnitine/choline ABC transporter	1.03	4.80E-02
malG	Maltose/maltodextrin ABC transporter, MalG permease	1.03	1.42E-02
ftsX	Cell division protein FtsX	1.03	1.42E-02
mtlA	PTS system mannitol-specific EIICB component	1.03	2.38E-01
SMU_943c	Hydroxymethylglutaryl-CoA synthase	1.03	5.47E-03
SMU_1867c	Alcohol dehydrogenase	1.03	8.35E-03
secY	Protein translocase subunit SecY	1.03	3.39E-02
SMU_1402c	Uncharacterized protein	1.03	4.37E-03
rnj	Ribonuclease J	1.03	2.57E-02
pyrC	Dihydroorotase	1.03	4.85E-03
atpG	ATP synthase gamma chain	1.03	1.63E-02
mapZ	Mid-cell-anchored protein Z	1.03	1.06E-02
purL	Phosphoribosylformylglycinamidine synthase, (FGAM synthase)	1.03	4.37E-03
rnj	Ribonuclease J	1.02	2.71E-02
SMU_2066c	Transmembrane protein	1.01	3.89E-02
fni	Isopentenyl-diphosphate delta-isomerase	1.01	1.51E-02
uppP	Undecaprenyl-diphosphatase	1.01	3.00E-02
SMU_1615c	DinB-like domain-containing protein	1.01	1.63E-02
SMU_1046c	GTP pyrophosphokinase	1.00	8.93E-03
hup	DNA-binding protein HU	1.00	3.58E-03

SMU_1293c	FeS assembly protein SufB	1.00	8.90E-03
alsS	Alpha-acetolactate synthase	1.00	1.05E-02
adcA	Surface adhesin AdcA protein-like protein putative Zn-binding lipoprotein	1.00	3.50E-02
dapA	4-hydroxy-tetrahydrodipicolinate synthase	1.00	5.58E-03
SMU_530c	Integral membrane protein	1.00	9.13E-02
SMU_2140c	Cyclic-di-AMP phosphodiesterase	1.00	1.44E-02
pepP	Aminopeptidase P	1.00	2.45E-03
SMU_993	Ribosome biogenesis GTPase A	-1.00	1.09E-02
SMU_1511c	Acetyltransferase	-1.00	4.27E-02
SMU_1717c	dITP/XTP pyrophosphatase	-1.00	4.94E-02
SMU_668c	Ribonucleoside-diphosphate reductase	-1.01	1.63E-02
tdk	Thymidine kinase	-1.01	1.18E-02
SMU_804	FRG domain-containing protein	-1.02	8.92E-03
thiD	pyridoxal kinase	-1.02	4.19E-02
SMU_100	Sorbose PTS system, IIB component	-1.02	6.40E-02
SMU_1141c	UPF0223 protein SMU_1141c	-1.02	3.39E-02
SMU_659	Response regulator SpaR	-1.03	1.74E-02
aroB	3-dehydroquinate synthase	-1.04	1.08E-02
aroK	Shikimate kinase	-1.04	7.01E-02
argB	Acetylglutamate kinase	-1.06	1.79E-02
SMU_646	Phosphatase	-1.06	4.89E-02
SMU_582	Farnesyl diphosphate synthase	-1.06	9.63E-03
cobQ	Lipid II isoglutaminyl synthase (glutamine-hydrolyzing) subunit GatD	-1.07	2.12E-02
smc	Chromosome partition protein Smc	-1.07	4.09E-03
SMU_2121c	YCII-related domain-containing protein	-1.08	4.05E-02
rplE	Large ribosomal subunit protein uL5	-1.08	4.71E-03

SMU_1406c	NADPH-dependent FMN reductase-like domain-containing protein	-1.08	1.36E-02
mnmG	tRNA uridine 5-carboxymethylaminomethyl modification enzyme MnmG	-1.08	4.37E-03
cas2	CRISPR-associated endoribonuclease Cas2	-1.09	6.31E-02
SMU_899	DegV domain-containing protein	-1.09	2.73E-02
alaS	Alanine--tRNA ligase	-1.09	8.93E-03
aspG	L-asparaginase	-1.09	1.20E-02
rplN	Large ribosomal subunit protein uL14	-1.10	5.08E-03
SMU_1475c	Cystathionine beta-lyase	-1.10	3.34E-02
ppaC	Probable manganese-dependent inorganic pyrophosphatase	-1.10	3.70E-02
SMU_1557c	CBS domain-containing protein	-1.10	3.52E-03
SMU_1300c	Cupin type-2 domain-containing protein	-1.10	2.45E-02
SMU_1108c	Uncharacterized protein	-1.11	3.47E-02
SMU_415	Aminoglycoside phosphotransferase domain-containing protein	-1.11	6.58E-03
SMU_1027	Transcription regulator	-1.11	3.07E-02
SMU_406c	Hydrolase	-1.12	1.66E-02
SMU_1306c	Nucleotide-binding protein SMU_1306c	-1.12	7.34E-03
glmM	Phosphoglucosamine mutase	-1.12	2.85E-02
SMU_2049c	Ribosomal RNA small subunit methyltransferase E	-1.12	1.32E-02
SMU_1028	Hydrolase or acyltransferase	-1.12	4.43E-03
trpB	Tryptophan synthase beta chain	-1.13	5.35E-03
galR	Galactose operon repressor GalR	-1.13	7.07E-03
prmA	Ribosomal protein L11 methyltransferase	-1.13	2.47E-02
SMU_1083c	L-threonylcarbamoyladenylate synthase	-1.14	7.43E-02
recN	DNA repair protein RecN	-1.14	4.37E-03
hemN	Heme chaperone HemW	-1.14	2.04E-02
SMU_181	Mevalonate kinase	-1.15	5.02E-02

SMU_516	Uncharacterized protein	-1.15	2.52E-02
SMU_1054	Glutamine amidotransferase	-1.15	2.12E-02
lplA	lipoate--protein ligase	-1.15	2.02E-02
lytR	Sensory transduction protein LytR	-1.16	1.18E-02
holB	DNA polymerase III, delta subunit	-1.16	7.42E-03
SMU_2027	Transcriptional regulator	-1.16	9.63E-02
SMU_1485c	Endonuclease	-1.16	4.71E-03
SMU_428	Uncharacterized protein	-1.16	3.55E-02
SMU_1008	Response regulator	-1.17	5.20E-02
SMU_1453c	Uncharacterized protein	-1.17	6.04E-02
SMU_1245c	Hydrolase	-1.18	5.12E-02
lacR	Lactose phosphotransferase system repressor	-1.18	4.37E-03
ung	Uracil-DNA glycosylase	-1.18	2.57E-02
SMU_1999c	Uncharacterized protein	-1.19	2.32E-02
SMU_561c	Hydrolase (MutT family)	-1.19	5.35E-02
udk	Uridine kinase	-1.19	1.21E-02
ilvE	Branched-chain-amino-acid aminotransferase	-1.19	3.70E-02
rimP	Ribosome maturation factor RimP	-1.19	2.11E-02
SMU_1151c	Hemerythrin-like domain-containing protein	-1.19	4.71E-03
alr	Alanine racemase	-1.19	2.26E-02
citZ	Citrate synthase	-1.20	2.43E-03
rgpD	Polysaccharide ABC transporter, ATP-binding protein	-1.20	5.69E-03
tmk	Thymidylate kinase	-1.20	2.22E-02
pyrK	Dihydroorotate dehydrogenase B (NAD(+)), electron transfer subunit	-1.21	2.41E-02
lysS	Lysine--tRNA ligase	-1.21	5.58E-03
rpsE	Small ribosomal subunit protein uS5	-1.21	5.31E-03
lepA	Elongation factor 4	-1.21	2.01E-02
SMU_1278c	Hydrolase	-1.21	3.15E-02

SMU_776	Methyltransferase	-1.22	2.41E-02
SMU_1484c	McrBC 5-methylcytosine restriction system component	-1.22	4.04E-03
metC	cysteine-S-conjugate beta-lyase	-1.23	1.42E-02
SMU_1773c	SMEK domain-containing protein	-1.23	6.32E-03
rpmJ	Large ribosomal subunit protein bL36	-1.23	2.97E-01
SMU_156	SseB protein N-terminal domain-containing protein	-1.23	2.07E-02
mutY	Adenine DNA glycosylase	-1.24	1.06E-02
tpx	Thiol peroxidase	-1.24	7.74E-02
recJ	Single-stranded-DNA-specific exonuclease RecJ	-1.24	1.42E-02
mrnC	Mini-ribonuclease 3	-1.24	1.65E-02
metK	S-adenosylmethionine synthase	-1.25	1.82E-02
SMU_1009	histidine kinase	-1.25	6.42E-03
SMU_1237c	SnoaL-like domain-containing protein	-1.25	3.47E-02
asnS	Asparagine--tRNA ligase	-1.25	2.43E-03
mutS	DNA mismatch repair protein MutS	-1.26	5.17E-03
ampM	Methionine aminopeptidase	-1.26	2.61E-02
metS	Methionine--tRNA ligase	-1.27	1.07E-02
glyQ	Glycine--tRNA ligase alpha subunit	-1.27	3.62E-03
SMU_1950	Pseudouridine synthase	-1.27	1.08E-02
gcrR	Response regulator GcrR for glucan-binding protein C	-1.27	3.52E-03
covS	histidine kinase	-1.28	1.32E-03
fmt	Methionyl-tRNA formyltransferase	-1.28	9.80E-03
SMU_1964c	Response regulator	-1.29	2.91E-02
pnp	Polyribonucleotide nucleotidyltransferase	-1.29	4.71E-03
rplB	Large ribosomal subunit protein uL2	-1.29	3.89E-03
SMU_1803c	Phosphoribosylglycinamide synthetase	-1.30	2.80E-03
gtfD	Glucosyltransferase-S	-1.30	1.41E-01
SMU_952	Methyltransferase	-1.31	2.50E-02

SMU_761	Protease	-1.32	1.98E-02
SMU_2126c	Uridine phosphorylase	-1.32	3.30E-02
SMU_1392c	Acetyltransferase	-1.32	2.03E-02
truB	tRNA pseudouridine synthase B	-1.33	1.98E-02
SMU_296	Ketopantoate reductase N-terminal domain-containing protein	-1.33	1.05E-02
prfC	Peptide chain release factor 3	-1.33	1.52E-02
fruR	FruR	-1.33	6.32E-03
rqcH	Rqc2 homolog RqcH	-1.34	7.09E-03
SMU_1487	Cobalamin-independent methionine synthase MetE C-terminal/archaeal domain-containing protein	-1.35	6.57E-03
rplA	Large ribosomal subunit protein uL1	-1.35	6.44E-03
dnaX	DNA-directed DNA polymerase	-1.35	6.89E-03
SMU_1417c	Oleoyl-acyl carrier protein thioesterase	-1.36	1.40E-02
covR	Transcriptional regulatory protein WalR	-1.36	1.63E-02
cah	Carbonic anhydrase	-1.36	4.68E-02
rpsZ	Small ribosomal subunit protein uS14	-1.36	8.09E-03
rnc	Ribonuclease 3	-1.36	1.56E-02
rsmH	Ribosomal RNA small subunit methyltransferase H	-1.37	5.58E-03
SMU_694c	Ferredoxin (4Fe-4S)	-1.38	1.25E-02
dnaA	Chromosomal replication initiator protein DnaA	-1.38	6.77E-03
rnhB	Ribonuclease HII	-1.38	3.14E-02
SMU_997	Inorganic ion ABC transporter, ATP-binding protein possible ferrichrome transport system	-1.38	6.42E-03
SMU_1301c	Methyltransferase	-1.38	1.52E-02
SMU_1801c	GTP-binding protein	-1.38	4.09E-03
SMU_852	Transcriptional regulator CpsY-like protein	-1.39	1.54E-02
SMU_440	Polyketide cyclase	-1.39	3.75E-02
msrA	Peptide methionine sulfoxide reductase MsrA	-1.40	2.22E-02

dfrA	Dihydrofolate reductase	-1.40	3.04E-02
SMU_1140c	Myo-inositol-1(Or 4)-monophosphatase	-1.40	1.68E-02
scnK	histidine kinase	-1.41	1.45E-03
rpsD	Small ribosomal subunit protein uS4	-1.41	7.32E-03
secA	Protein translocase subunit SecA	-1.42	5.69E-03
uppS	Isoprenyl transferase	-1.42	8.00E-03
htrA	Serine protease HtrA	-1.42	3.07E-02
dnaI	DNA replication protein primosome component (Helicase loader)	-1.42	8.90E-03
rimM	Ribosome maturation factor RimM	-1.43	9.41E-03
SMU_1925c	DUF177 domain-containing protein	-1.43	1.36E-02
mutL	DNA mismatch repair protein MutL	-1.44	5.58E-03
SMU_399	C3-degrading proteinase	-1.45	4.68E-02
SMU_1081c	Pseudouridylate synthases, 23S RNA-specific	-1.45	7.07E-03
mleR	Transcriptional regulator	-1.45	1.27E-02
SMU_897	Type I restriction enzyme endonuclease subunit	-1.45	6.89E-03
SMU_954	pyridoxal kinase	-1.47	2.59E-02
clpX	ATP-dependent Clp protease ATP-binding subunit ClpX	-1.48	1.18E-02
trmB	tRNA (guanine-N(7)-)-methyltransferase	-1.48	8.18E-03
SMU_488	peptidylprolyl isomerase	-1.48	2.10E-02
SMU_723	Calcium-transporting ATPase P-type ATPase	-1.49	4.71E-03
dnaG	DNA primase	-1.50	6.42E-03
SMU_848	Ribosomal processing cysteine protease Prp	-1.50	1.66E-02
SMU_1945	VTC domain-containing protein	-1.50	3.88E-02
dam	Site-specific DNA-methyltransferase (adenine-specific)	-1.50	1.36E-02
SMU_392c	BaiN-like insert domain-containing protein	-1.50	1.16E-02
SMU_2137c	CAAX amino protease family protein	-1.50	2.24E-02
SMU_1645	Tellurite resistance protein	-1.50	2.41E-02

abcX	Putative ABC transporter ATP-binding protein	-1.51	6.01E-03
SMU_1636c	ASCH domain-containing protein	-1.51	3.62E-02
rpsC	Small ribosomal subunit protein uS3	-1.51	3.49E-03
metB	Cystathionine gamma-synthase possible bifunctional enzyme	-1.52	5.35E-03
pth	Peptidyl-tRNA hydrolase	-1.52	1.40E-02
dnaC	Replicative DNA helicase	-1.53	2.45E-03
murC	UDP-N-acetylmuramate--L-alanine ligase	-1.53	2.33E-02
SMU_1125c	Methyltransferase small domain-containing protein	-1.53	5.71E-02
murF	UDP-N-acetylmuramoyl-tripeptide--D-alanyl-D-alanine ligase	-1.53	3.19E-02
SMU_320	5-formyltetrahydrofolate cyclo-ligase	-1.55	3.34E-02
polC	DNA polymerase III PolC-type	-1.55	8.35E-03
murB	UDP-N-acetylenolpyruvoylglucosamine reductase	-1.55	1.64E-02
trpA	Tryptophan synthase alpha chain	-1.56	1.65E-02
rsgA	Small ribosomal subunit biogenesis GTPase RsgA	-1.56	3.52E-03
serB	phosphoserine phosphatase	-1.57	5.27E-03
SMU_187c	tRNA-dihydrouridine synthase	-1.57	2.12E-02
ilvA	L-threonine dehydratase	-1.57	6.81E-03
SMU_508	Hydrolase	-1.57	7.98E-03
apbE	FAD:protein FMN transferase	-1.57	1.32E-02
gltB	NADPH-dependent glutamate synthase (Small subunit)	-1.58	7.56E-03
SMU_470	UPF0398 protein SMU_470	-1.58	5.81E-02
fabD	Malonyl CoA-acyl carrier protein transacylase	-1.58	1.57E-02
hisF	Imidazole glycerol phosphate synthase subunit HisF	-1.59	3.44E-03
SMU_854	Pseudouridine synthase	-1.60	1.18E-02
aroA	3-phosphoshikimate 1-carboxyvinyltransferase	-1.60	1.53E-02
bipA	Large ribosomal subunit assembly factor BipA	-1.61	1.68E-02
SMU_700c	Phosphoglycerate mutase-like protein	-1.61	5.08E-03

rmlA	Glucose-1-phosphate thymidylyltransferase	-1.61	1.75E-02
aspB	Asparagine--oxo-acid transaminase	-1.62	1.52E-02
nrdI	Putative NrdI-like protein	-1.62	1.72E-02
recA	Protein RecA	-1.63	9.40E-03
rf2	Peptide chain release factor 2	-1.63	1.81E-02
SMU_497c	Xaa-Pro dipeptidase	-1.63	2.04E-02
deoC	Deoxyribose-phosphate aldolase	-1.64	1.08E-02
SMU_742	Hydrolase	-1.64	6.73E-03
murD	UDP-N-acetylmuramoylalanine--D-glutamate ligase	-1.64	2.14E-02
SMU_1789c	Probable transcriptional regulatory protein SMU_1789c	-1.64	2.10E-02
sapR2	Sakacin A production response regulator	-1.65	1.37E-02
rs1	Ribosomal protein S1 sequence specific DNA-binding protein	-1.67	8.90E-03
leuD	3-isopropylmalate dehydratase small subunit	-1.68	1.89E-02
tagI	3-methyl-adenine DNA glycosylase I	-1.68	4.68E-02
fusA	Elongation factor G	-1.70	9.90E-03
SMU_927	Response regulator	-1.71	3.70E-02
clpC	Class III stress response-related ATP-dependent Clp protease, ATP-binding subunit	-1.71	2.87E-03
der	GTPase Der	-1.71	9.31E-03
SMU_2120c	Putative 3-methyladenine DNA glycosylase	-1.72	5.33E-02
wapA	Wall-associated protein	-1.72	3.89E-03
aroC	Chorismate synthase	-1.73	6.95E-03
SMU_774	Hydrolase	-1.73	7.27E-03
thrC	Threonine synthase	-1.75	1.76E-02
mnmA	tRNA-specific 2-thiouridylase MnmA	-1.76	1.37E-02
SMU_2147c	LysM domain-containing protein	-1.76	4.37E-03
SMU_1431c	ABC transporter, ATP-binding protein	-1.77	6.68E-03
nusG	Transcription termination/antitermination protein NusG	-1.77	1.29E-02

folK	2-amino-4-hydroxy-6-hydroxymethyldihydropteridine diphosphokinase	-1.77	2.62E-02
radA	DNA repair protein RadA	-1.78	4.37E-03
SMU_61	Transcriptional regulator	-1.78	2.22E-02
SMU_850	N-acetyltransferase domain-containing protein	-1.79	2.48E-02
amyA	Intracellular alpha-amylase	-1.79	1.08E-02
SMU_1315c	ATP-binding protein	-1.81	5.08E-03
SMU_301	Nucleotidyltransferase family protein	-1.82	2.50E-02
folD	Bifunctional protein FolD	-1.82	1.52E-02
SMU_669c	Glutaredoxin-like protein NrdH	-1.82	1.52E-02
nusA	Transcription termination/antitermination protein NusA	-1.84	1.05E-02
SMU_65	protein-tyrosine-phosphatase	-1.86	2.72E-02
hemK	Release factor glutamine methyltransferase	-1.88	1.91E-02
ylmE	Pyridoxal phosphate homeostasis protein	-1.88	1.24E-02
tuf	Elongation factor Tu	-1.88	2.11E-02
cysK	Cysteine synthase	-1.90	8.90E-03
SMU_1347c	ABC3 transporter permease C-terminal domain-containing protein	-1.91	3.00E-06
hpt	Hypoxanthine-guanine phosphoribosyltransferase	-1.93	1.65E-02
srtA	Sortase	-1.94	1.68E-02
SMU_721	DUF1934 domain-containing protein	-1.95	3.20E-02
SMU_1209c	Uncharacterized protein	-1.95	1.21E-02
nrdD	Anaerobic ribonucleoside-triphosphate reductase	-1.96	1.37E-02
SMU_1747c	Phosphatase	-1.96	1.21E-02
rlmN	Probable dual-specificity RNA methyltransferase RlmN	-1.96	9.60E-03
prfA	Peptide chain release factor 1	-1.97	1.37E-02
hisC	Histidinol-phosphate aminotransferase	-2.00	6.81E-03
acn	Aconitate hydratase A	-2.01	9.63E-03
exoA	Exodeoxyribonuclease III	-2.03	1.66E-02

SMU_1483c	N-acetyltransferase domain-containing protein	-2.04	2.19E-02
SMU_816	Aminotransferase	-2.09	1.52E-02
rlmH	Ribosomal RNA large subunit methyltransferase H	-2.09	3.58E-03
SMU_937	diphosphomevalonate decarboxylase	-2.10	1.65E-02
SMU_401c	N-acetyltransferase domain-containing protein	-2.10	1.54E-02
SMU_1772c	Uncharacterized protein	-2.12	1.99E-02
leuC	3-isopropylmalate dehydratase large subunit	-2.13	1.62E-02
trpD	Anthranilate phosphoribosyltransferase	-2.14	9.78E-03
aroE	Shikimate dehydrogenase (NADP(+))	-2.14	1.44E-02
SMU_1774c	Aldose 1-epimerase	-2.14	2.57E-02
SMU_751	S1 motif domain-containing protein	-2.14	9.38E-03
SMU_1349	Transcriptional regulator	-2.15	2.84E-03
folP	Dihydropteroate synthase	-2.15	2.42E-02
obg	GTPase Obg	-2.18	1.44E-02
SMU_420	Ribosomal protein	-2.22	1.68E-02
SMU_1348c	ABC transporter ATP-binding protein	-2.23	1.54E-04
ychF	Ribosome-binding ATPase YchF	-2.26	1.65E-02
SMU_326	Isochorismatase-like domain-containing protein	-2.29	1.42E-02
SMU_630	Beta-lactamase class A catalytic domain-containing protein	-2.30	2.16E-02
SMU_1721c	Diaminopimelate decarboxylase	-2.33	1.68E-02
SMU_2129c	MIP18 family-like domain-containing protein	-2.35	2.29E-02
SMU_984	Peptidase C51 domain-containing protein	-2.43	2.76E-02
murA1	UDP-N-acetylglucosamine 1-carboxyvinyltransferase 1	-2.43	1.77E-02
queA	S-adenosylmethionine:tRNA ribosyltransferase-isomerase	-2.45	1.23E-02
hisH	Imidazole glycerol phosphate synthase subunit HisH	-2.49	5.99E-03
bacD	Bacitracin synthetase	-2.54	2.04E-04
SMU_1344c	[acyl-carrier-protein] S-malonyltransferase	-2.66	9.16E-05

murA2	UDP-N-acetylglucosamine 1-carboxyvinyltransferase 2	-2.67	1.41E-02
SMU_1205c	HNH endonuclease	-2.76	1.51E-02
pknB	non-specific serine/threonine protein kinase	-2.93	4.32E-07
SMU_1343c	Polyketide synthase	-3.32	9.70E-04
bacA1	Bacitracin synthetase 1 BacA	-3.48	4.52E-04
bacT	Thioesterase BacT	-3.48	2.84E-03
bacA2	Surfactin synthetase	-3.53	5.48E-04
SMU_1345c	Peptide synthetase	-3.68	9.71E-04
SMU_1341c	Gramicidin S synthetase	-3.72	5.48E-04
ssb2	Single-stranded DNA-binding protein	-3.77	6.31E-04
SMU_483	Phosphoprotein phosphatase (PppL protein)	-4.95	4.60E-08

Table S4 Phosphoproteomics comparison of *S. mutans* UA159 vs *S. mutans* Δ*pknB*

Gene	Description	Phosphorylation site	Phospho log2FC	adj.P.Val	Protein log2FC
aspB	Asparagine--oxo-acid transaminase	S242	0.68	0.43	0.28
		T244	0.68	0.43	0.28
divIVA	Cell division protein DivIVA	T232	ABSENT	NA	0.33
		T262	ABSENT	NA	0.33
eno	Enolase	T5	PRESENT	NA	1.83
ffh	Signal recognition particle protein	S397	0.02	0.94	0.40
fruA	Fructan beta-fructosidase	S1358	-0.23	0.71	-1.78
		S1355	1.25	0.02	-1.78
		S1356	1.25	0.02	-1.78
fruI	Inducible fructose permease	S279	-0.56	0.25	-0.18
gapC	Glyceraldehyde-3-phosphate dehydrogenase	S212	0.78	0.15	0.26
gpsB	Cell cycle protein GpsB	S3	PRESENT	NA	0.34
groES	Co-chaperonin GroES	T20	0.16	0.79	-0.07
mapZ	Mid-cell-anchored protein Z	T127	-10.18	0.00	0.41
		T18	ABSENT	NA	0.41
mltG	Endolytic murein transglycosylase	T191	-8.65	0.00	-0.11
		T102	-5.91	0.00	-0.11
		T166	ABSENT	NA	-0.11
ptsH	Phosphocarrier protein HPr	T12	-0.70	0.40	-2.99
pttB	PTS system, trehalose-specific IIABC component	S491	-0.04	0.88	0.18
		S493	0.13	0.71	0.18
		T487	0.80	0.01	0.18
rgpA	RgpAc glycosyltransferase	S214	-0.98	0.01	0.19
rpmF	Large ribosomal subunit protein bL32	T32	-5.16	0.00	-0.58
rpsM	Small ribosomal subunit protein uS13	S50	0.39	0.14	0.20
rs1	Ribosomal protein S1 sequence specific DNA-binding protein	T198	-0.45	0.34	0.20
scnK	histidine kinase	S413	1.46	0.00	-0.40

SMU_104	Alpha-glucosidase glycosyl hydrolase	S87	0.16	0.74	-0.03
		S88	0.16	0.74	-0.03
SMU_1143c	Riboflavin biosynthesis protein	T245	1.98	0.00	0.68
SMU_1641c	CsbD-like domain-containing protein	T29	-1.88	0.00	-1.27
SMU_1772c	Uncharacterized protein	Y55	2.84	0.00	-0.48
SMU_1787c	Secreted protein	S123	-0.68	0.08	-0.55
levX	PTS fructose transporter subunit IA	Y11	-2.45	0.00	-0.16
levF	PTS system, mannose-specific IIC component	S269	0.26	0.71	-0.30
levE	PTS system, mannose-specific IIB component	T17	-0.19	0.76	-1.08
levD	PTS system, sugar-specific enzyme IIA component	S135	-1.11	0.08	-1.51
pgfM2	Transmembrane protein	T16	ABSENT	NA	-0.55
ireB-like	UPF0297 protein SMU_2079c	T7	-7.07	0.00	0.47
		T4	-5.37	0.00	0.47
tsf	Elongation factor Ts	T5	4.74	0.00	-0.49
tuf	Elongation factor Tu	S52	-0.63	0.03	-0.26

Table S5 Phosphoproteomics comparison of *S. mutans* UA159 vs *S. mutans* Δ*pppL*

Gene	Description	Phosphorylation site	Phospho logFC	adj.P.Val	Protein logFC
adhE	Aldehyde-alcohol dehydrogenase	S22	PRESENT	NA	1.72
		T7	0.08	0.92	1.72
argR	Arginine repressor	T40	-0.38	0.56	0.36
bipA	Large ribosomal subunit assembly factor BipA	T559	7.89	0.10	-1.61
cysE	Serine acetyltransferase	T193	1.55	0.11	0.77
cysK	Cysteine synthase	S120	-0.54	0.31	-1.90
divIVA	Cell division protein DivIVA	S84	0.71	0.38	0.63
		T195	-0.07	0.95	0.63
		T201	-0.33	0.75	0.63
		T262	2.18	0.17	0.63
		T4	0.18	0.92	0.63
		T77	-1.54	0.00	0.63
dnaK	Chaperone protein DnaK	T430	PRESENT	NA	0.32
		Y104	PRESENT	NA	0.32
efp	Elongation factor P	S184	0.23	0.70	-0.29
		T145	1.27	0.54	-0.29
eno	Enolase	T200	-0.26	0.80	0.83
fbaA	Fructose-bisphosphate aldolase	T233	1.15	0.11	0.63
		Y257	0.36	0.71	0.63
ffh	Signal recognition particle protein	S397	-0.02	0.95	-0.14
		T285	0.48	0.75	-0.14
folC	tetrahydrofolate synthase	S84	0.08	0.92	-0.82
frr	Ribosome-recycling factor	S19	0.22	0.88	0.20
fruA	Fructan beta-fructosidase	S1355	-0.54	0.32	-0.47
		S1356	-0.54	0.32	-0.47

		S1358	-0.54	0.32	-0.47
fruC	Constitutive fructose permease	S114	0.48	0.59	2.20
		T121	PRESENT	NA	2.20
fruI	Inducible fructose permease	S275	PRESENT	NA	-0.07
		S278	0.23	0.85	-0.07
		S282	PRESENT	NA	-0.07
		S283	PRESENT	NA	-0.07
		S291	-0.06	0.95	-0.07
		S30	-0.62	0.75	-0.07
		T55	0.36	0.71	-0.07
ftsZ	Cell division protein FtsZ	S333	0.00	0.99	-0.88
		S4	0.63	0.39	-0.88
fusA	Elongation factor G	S480	1.73	0.12	-1.70
		S638	-1.70	0.08	-1.70
		T238	-2.14	0.01	-1.70
gapC	Glyceraldehyde-3-phosphate dehydrogenase	S212	0.55	0.64	0.87
		Y331	PRESENT	NA	0.87
glgC	Glucose-1-phosphate adenylyltransferase	S170	2.00	0.02	1.19
glk	Glucokinase	S210	PRESENT	NA	0.57
		S283	PRESENT	NA	0.57
glyA	Serine hydroxymethyltransferase	Y55	1.16	0.17	0.49
gpsB	Cell cycle protein GpsB	S3	PRESENT	NA	0.41
		T72	3.65	0.04	0.41
		T73	0.87	0.46	0.41
		T87	1.58	0.15	0.41

		T88	1.63	0.10	0.41
		S75	PRESENT	NA	0.41
		T72	0.63	0.64	0.41
greA	Transcription elongation factor GreA	S40	-0.22	0.80	0.26
		S45	0.38	0.56	0.26
		S48	0.04	0.95	0.26
		S79	PRESENT	NA	0.26
groEL	Chaperonin GroEL	S356	PRESENT	NA	0.71
		S460	-0.22	0.64	0.71
		T25	3.19	0.12	0.71
		T268	0.32	0.77	0.71
		T355	-0.08	0.91	0.71
		T385	0.14	0.92	0.71
		T430	PRESENT	NA	0.71
groES	Co-chaperonin GroES	S30	-1.05	0.18	0.69
		T20	PRESENT	NA	0.69
gshAB	Glutathione biosynthesis bifunctional protein GshAB	T210	PRESENT	NA	0.03
gtfB	Glucosyltransferase-I	S1272	-0.12	0.93	2.15
		T1041	0.30	0.64	2.15
		T119	PRESENT	NA	2.15
gtfC	Glucosyltransferase-SI	T990	-3.49	0.00	-0.14
hisS	Histidine--tRNA ligase	T57	-1.22	0.25	0.10
hprK	HPr kinase/phosphorylase	T300	PRESENT	NA	0.44
hup	DNA-binding protein HU	T18	PRESENT	NA	1.00

		T66	0.25	0.80	1.00
ilvC	Ketol-acid reductoisomerase (NADP(+))	S99	0.71	0.20	-0.65
khpB	RNA-binding protein KhpB	T88	3.65	0.01	0.18
ldh	L-lactate dehydrogenase	T227	1.50	0.01	-0.04
lemA	Cytoplasmic membrane protein LemA-like protein	T170	-0.04	0.95	1.37
lepA	Elongation factor 4	S41	-0.18	0.85	-1.21
		S42	0.22	0.79	-1.21
		T39	0.40	0.56	-1.21
lytS	Sensor protein LytS	T524	-0.23	0.84	0.53
mapZ	Mid-cell-anchored protein Z	S22	0.69	0.34	1.03
		T101	-0.33	0.75	1.03
		T127	-0.93	0.07	1.03
		T143	PRESENT	NA	1.03
		T18	0.19	0.78	1.03
		T2	PRESENT	NA	1.03
mltG	Endolytic murein transglycosylase	T102	-2.44	0.00	0.45
		T105	3.37	0.00	0.45
		T107	1.27	0.10	0.45
		T166	-0.33	0.83	0.45
		T191	0.49	0.50	0.45
		T222	2.23	0.11	0.45
		T41	PRESENT	NA	0.45
		T102	1.68	0.05	0.45
		T105	1.68	0.05	0.45
		S375	-0.06	0.95	0.45
		T374	-0.06	0.95	0.45
		T379	-0.06	0.95	0.45
murD	UDP-N-acetylmuramoylalanine--D-glutamate ligase	T332	0.15	0.80	-1.64

pepC	Aminopeptidase	T7	PRESENT	NA	0.57
pepN	Aminopeptidase	S606	PRESENT	NA	0.64
pepX	Xaa-Pro dipeptidyl-peptidase	S574	-0.25	0.85	0.70
pfkA	ATP-dependent 6-phosphofructokinase	S159	0.34	0.32	0.69
pgi	Glucose-6-phosphate isomerase	T148	PRESENT	NA	0.74
		T149	-0.32	0.52	0.74
polC	DNA polymerase III PolC-type	T190	-0.31	0.85	-1.55
proS	Proline--tRNA ligase	S286	PRESENT	NA	-0.30
ptnA	PTS system mannose-specific EIIAB component	T145	1.55	0.02	0.01
		T161	PRESENT	NA	0.01
		T203	0.94	0.15	0.01
ptnC	PTS system, mannose-specific component IIC	S261	0.27	0.92	1.21
ptsH	Phosphocarrier protein HPr	S27	1.84	0.15	0.36
		T12	1.28	0.19	0.36
		Y37	PRESENT	NA	0.36
pttB	PTS system, trehalose-specific IIABC component	S491	-0.38	0.38	0.32
		S493	0.32	0.64	0.32
		S653	-0.56	0.32	0.32
		T29	1.02	0.10	0.32
		T485	-0.68	0.19	0.32
		T487	-0.32	0.85	0.32
		T503	0.09	0.92	0.32
ptxA	Ascorbate-specific PTS system EIIA component	Y153	PRESENT	NA	0.98
ptxB	PTS system, enzyme IIB component	S40	2.25	0.29	1.17
pykF	Pyruvate kinase	T412	0.15	0.91	0.00

pyrG	CTP synthase	S451	PRESENT	NA	-0.46
pyrH	Uridylate kinase	S14	0.61	0.28	0.23
queF	NADPH-dependent 7-cyano-7-deazaguanine reductase	T10	PRESENT	NA	0.34
recA	Protein RecA	S175	-2.88	0.00	-1.63
rf2	Peptide chain release factor 2	T16	-0.37	0.59	-1.63
rnz	Ribonuclease Z	T162	1.14	0.27	0.91
rpiA	Ribose-5-phosphate isomerase A	S55	PRESENT	NA	0.47
rplI	Large ribosomal subunit protein bL9	S76	-0.60	0.54	0.07
rplO	Large ribosomal subunit protein uL15	S131	0.88	0.44	-0.96
rplR	Large ribosomal subunit protein uL18	S89	-0.05	0.95	-0.57
		T69	1.50	0.11	-0.57
rplW	Large ribosomal subunit protein uL23	T89	0.40	0.69	-0.98
rplX	Large ribosomal subunit protein uL24	S66	PRESENT	NA	0.86
rpmA	Large ribosomal subunit protein bL27	S46	PRESENT	NA	0.01
		T41	PRESENT	NA	0.01
rpmC	Large ribosomal subunit protein uL29	T47	2.79	0.04	0.52
rpmF	Large ribosomal subunit protein bL32	S37	5.23	0.04	0.66
		T32	0.05	0.92	0.66
rpoA	DNA-directed RNA polymerase subunit alpha	S279	-1.52	0.15	-0.04
		S281	-1.52	0.15	-0.04
rpsH	Small ribosomal subunit protein uS8	S23	0.32	0.79	-0.74
rpsJ	Small ribosomal subunit protein uS10	S35	PRESENT	NA	-0.91
rpsM	Small ribosomal subunit protein uS13	S50	-1.31	0.05	-0.07
rpsR	Small ribosomal subunit protein bS18	T31	1.39	0.05	-0.88
rpsU	Small ribosomal subunit protein bS21	S11	1.32	0.55	1.04
rs1	Ribosomal protein S1 sequence specific DNA-binding protein	T198	-1.29	0.05	-1.67

		T338	-0.74	0.29	-1.67
secY	Protein translocase subunit SecY	Y347	PRESENT	NA	1.03
sepF	Cell division protein SepF	S41	-0.35	0.79	0.26
		S71	PRESENT	NA	0.26
		S78	0.68	0.29	0.26
SMU_1037c	histidine kinase	Y139	-2.16	0.02	0.69
SMU_104	Alpha-glucosidase glycosyl hydrolase	S87	-1.08	0.10	-0.24
		S88	-1.08	0.10	-0.24
SMU_1081c	Pseudouridylate synthases, 23S RNA-specific	T245	PRESENT	NA	-1.45
SMU_1120	Sugar ABC transporter, ATP-binding protein	T245	PRESENT	NA	0.15
SMU_1208c	Uncharacterized protein	S158	-0.06	0.95	-0.54
SMU_1309c	Glycerol dehydrogenase	Y358	3.65	0.04	1.23
SMU_1621c	UPF0346 protein SMU_1621c	T11	-1.87	0.07	-0.39
		Y6	-1.87	0.07	-0.39
SMU_164	tRNA/rRNA methyltransferase	S65	0.41	0.39	-0.63
		T63	0.41	0.39	-0.63
SMU_1641c	CsbD-like domain-containing protein	T29	0.81	0.17	1.29
SMU_1644c	Uncharacterized protein	T35	PRESENT	NA	0.52
SMU_1681c	VOC domain-containing protein	S35	0.48	0.59	1.49
SMU_1722c	Integral membrane protein	T5	PRESENT	NA	1.98
SMU_1772c	Uncharacterized protein	Y55	0.75	0.56	-2.12
	Secreted protein	T118	0.78	0.13	1.11

SMU_1787c		T121	0.18	0.78	1.11
		T95	PRESENT	NA	1.11
SMU_1853	Integral membrane protein	T33	0.69	0.42	0.95
SMU_1876	DUF2975 domain-containing protein	S3	-2.36	0.04	1.26
		Y4	-2.36	0.04	1.26
SMU_1879	PTS system, mannose-specific component IID	S174	PRESENT	NA	1.29
SMU_1881c	ABC transporter, ATP-binding protein	S203	-1.91	0.11	ABSENT
		S207	-1.91	0.11	ABSENT
		T202	-1.91	0.11	ABSENT
SMU_1936c	DNA-directed RNA polymerase subunit delta	S128	PRESENT	NA	1.12
levX	PTS fructose transporter subunit IA	Y11	-1.25	0.20	0.10
levG	PTS system, mannose-specific IID component	T177	0.16	0.92	0.80
levF	PTS system, mannose-specific IIC component	S273	0.01	0.99	0.77
levE	PTS system, mannose-specific IIB component	T17	0.50	0.65	-0.35
levD	PTS system, sugar-specific enzyme IIA component	S135	-0.77	0.19	-0.03
		T48	0.47	0.57	-0.03
SMU_205c	Bacteriocin immunity protein	S64	ABSENT	NA	0.55
pgfM2	Transmembrane protein	T16	2.08	0.06	0.55
ireB-like	UPF0297 protein SMU_2079c	T4	-7.04	0.00	1.97
		T7	4.43	0.08	1.97
		T4	0.84	0.64	1.97
		T7	0.84	0.64	1.97
SMU_2127	Succinate semialdehyde dehydrogenase	S214	3.60	0.21	1.63
SMU_333	MFS transporter	T2	-0.30	0.79	0.97

SMU_38c	Transcriptional regulator	T41	0.99	0.30	ABSENT
SMU_393	Regulator of chromosome segregation-like C-terminal domain-containing protein	T130	PRESENT	NA	0.18
		T41	-1.05	0.26	0.18
SMU_447	UPF0291 protein SMU_447	T62	-1.28	0.06	0.97
SMU_470	UPF0398 protein SMU_470	Y144	-1.46	0.00	-1.58
		Y146	-1.46	0.00	-1.58
SMU_487	Response regulator	T131	-0.20	0.84	-0.29
SMU_501	Uncharacterized protein	S61	1.07	0.17	1.11
		Y70	PRESENT	NA	1.11
SMU_502	TcaA 4th domain-containing protein	S53	PRESENT	NA	-0.03
		T52	4.84	0.00	-0.03
SMU_530c	Integral membrane protein	S162	1.39	0.17	1.00
SMU_567	Glutamine ABC transporter, permease component	S4	-0.35	0.78	0.85
		S7	-0.35	0.78	0.85
SMU_635	Integral membrane protein	S103	0.25	0.69	0.72
SMU_695	LysM domain-containing protein	T15	-1.03	0.11	1.29
SMU_720	Probable membrane transporter protein	T157	1.25	0.25	1.38
SMU_752	Protein SprT-like	T4	-1.69	0.17	-0.73
		Y6	-1.69	0.17	-0.73
SMU_757	Gas vesicle protein	T76	-2.56	0.02	1.45
SMU_768c	YolD-like protein	S54	7.14	0.00	0.81
SMU_793	Thioesterase domain-containing protein	T84	PRESENT	NA	0.25
SMU_913	Glutamate dehydrogenase	T192	-1.48	0.10	0.30
sodA	Superoxide dismutase [Mn/Fe]	T123	0.62	0.30	0.27
		T202	0.41	0.75	0.27
thrC	Threonine synthase	T2	-0.39	0.64	-1.75

tig	Trigger factor	S385	0.27	0.79	-0.88
		S400	-0.12	0.85	-0.88
		T40	-1.30	0.07	-0.88
treA	Alpha,alpha-phosphotrehalase	S391	-1.23	0.03	-0.42
tuf	Elongation factor Tu	S390	0.11	0.85	-1.88
		S52	-0.87	0.12	-1.88
		T261	-1.67	0.06	-1.88
		T387	-0.78	0.12	-1.88
whiA	Probable cell division protein WhiA	T225	0.93	0.64	0.30

Table S6 Phosphorylated serine, threonine, or tyrosine residues uniquely identified in *S. mutans* Δ*pppL*

				Δ*pppL*					UA159				
**Gene**	**Protein Description**	**Phospho Site**	**LP***	**1**	**2**	**3**	**4**	**5**	**1**	**2**	**3**	**4**	**5**
adhE	Aldehyde-alcohol dehydrogenase	S22	0.94	10.8	11.1	11.2	10.7	NA	NA	NA	NA	NA	NA
dnaK	Chaperone protein DnaK	T430	1.00	10.5	10.7	10.8	11.0	NA	NA	NA	NA	NA	NA
dnaK	Chaperone protein DnaK	Y104	1.00	9.6	10.5	NA	10.1	NA	NA	NA	NA	NA	NA
fruC	Constitutive fructose permease	T121	0.99	11.0	10.8	11.0	10.8	10.9	NA	NA	NA	NA	NA
fruI	Inducible fructose permease	S275	0.97	10.6	11.0	10.1	10.2	NA	NA	NA	NA	NA	NA
fruI	Inducible fructose permease	S282	0.80	10.0	10.0	10.6	10.5	NA	NA	NA	NA	NA	NA
fruI	Inducible fructose permease	S283	0.82	10.3	10.7	10.6	10.8	NA	NA	NA	NA	NA	NA
gapC	Glyceraldehyde-3-phosphate dehydrogenase	Y331	1.00	11.0	10.8	11.0	11.4	NA	NA	NA	NA	NA	NA
glk	Glucokinase	S210	0.84	11.1	10.7	11.0	11.4	NA	NA	NA	NA	NA	NA
glk	Glucokinase	S283	1.00	10.7	10.0	10.9	10.6	NA	NA	NA	NA	NA	NA
gpsB	Cell cycle protein GpsB	S3	1.00	10.5	10.0	10.6	10.5	NA	NA	NA	NA	NA	NA
gpsB	Cell cycle protein GpsB	S75	0.76	11.2	11.3	11.1	10.9	10.0	NA	NA	NA	NA	NA
greA	Transcription elongation factor GreA	S79	1.00	10.6	10.4	10.5	10.1	NA	NA	NA	NA	NA	NA
groEL	Chaperonin GroEL	S356	0.77	11.1	11.0	11.1	10.8	NA	NA	NA	NA	NA	NA
groEL	Chaperonin GroEL	T430	1.00	10.4	10.8	10.8	10.6	NA	NA	NA	NA	NA	NA
groES	Co-chaperonin GroES	T20	1.00	10.9	11.0	10.8	10.9	12.4	NA	NA	NA	NA	NA
gshAB	Glutathione biosynthesis bifunctional protein GshAB	T210	0.99	9.7	9.9	10.2	10.5	NA	NA	NA	NA	NA	NA
gtfB	Glucosyltransferase-I	T119	0.94	10.9	10.2	10.8	10.3	NA	NA	NA	NA	NA	NA
hprK	HPr kinase/phosphorylase	T300	1.00	NA	9.8	10.3	10.6	NA	NA	NA	NA	NA	NA
hup	DNA-binding protein HU	T18	1.00	11.3	10.6	11.4	11.0	10.8	NA	NA	NA	NA	NA
mapZ	Mid-cell-anchored protein Z	T143	0.99	11.7	12.1	11.2	12.0	13.0	NA	NA	NA	NA	NA
mapZ	Mid-cell-anchored protein Z	T2	1.00	11.4	11.0	10.8	10.4	9.8	NA	NA	NA	NA	NA

mltG	Endolytic murein transglycosylase	T41	0.85	11.2	11.4	10.8	11.1	13.2	NA	NA	NA	NA	NA
pepC	Aminopeptidase	T7	0.97	10.1	10.2	9.7	10.0	NA	NA	NA	NA	NA	NA
pepN	Aminopeptidase	S606	0.88	10.8	10.1	10.5	10.7	NA	NA	NA	NA	NA	NA
pgi	Glucose-6-phosphate isomerase	T148	0.97	10.6	10.7	10.7	10.8	NA	NA	NA	NA	NA	NA
proS	Proline--tRNA ligase	S286	1.00	10.2	9.8	10.1	9.9	NA	NA	NA	NA	NA	NA
ptnA	PTS system mannose-specific EIIAB component	T161	1.00	12.6	12.4	12.9	12.4	12.8	NA	NA	NA	NA	NA
ptsH	Phosphocarrier protein HPr	Y37	1.00	9.6	10.3	9.5	10.6	NA	NA	NA	NA	NA	NA
ptxA	Ascorbate-specific PTS system EIIA component	Y153	0.98	10.4	10.2	10.8	10.3	NA	NA	NA	NA	NA	NA
pyrG	CTP synthase	S451	0.80	10.1	9.6	10.1	10.0	NA	NA	NA	NA	NA	NA
queF	NADPH-dependent 7-cyano-7-deazaguanine reductase	T10	1.00	10.6	11.2	9.5	10.7	12.0	NA	NA	NA	NA	NA
rpiA	Ribose-5-phosphate isomerase A	S55	0.87	10.0	10.3	10.3	9.8	NA	NA	NA	NA	NA	NA
rplX	Large ribosomal subunit protein uL24	S66	1.00	9.8	10.2	10.4	10.3	NA	NA	NA	NA	NA	NA
rpmA	Large ribosomal subunit protein bL27	S46	0.99	10.9	10.1	9.0	10.7	NA	NA	NA	NA	NA	NA
rpmA	Large ribosomal subunit protein bL27	T41	1.00	11.3	11.1	10.7	11.1	12.3	NA	NA	NA	NA	NA
rpsJ	Small ribosomal subunit protein uS10	S35	0.99	10.0	10.2	10.6	10.2	NA	NA	NA	NA	NA	NA
secY	Protein translocase subunit SecY	Y347	1.00	10.7	9.9	10.3	10.2	NA	NA	NA	NA	NA	NA
sepF	Cell division protein SepF	S71	1.00	10.4	NA	9.6	10.3	NA	NA	NA	NA	NA	NA
SMU_1081c	Pseudouridylate synthases, 23S RNA-specific	T245	0.82	9.2	NA	NA	9.1	12.5	NA	NA	NA	NA	NA
SMU_1120	Sugar ABC transporter, ATP-binding protein	T245	0.98	10.4	10.5	10.7	11.0	12.5	NA	NA	NA	NA	NA

SMU_1644c	Uncharacterized protein	T35	1.00	11.6	11.2	11.8	11.1	NA	NA	NA	NA	NA	NA
SMU_1722c	Integral membrane protein	T5	1.00	11.0	11.3	11.2	11.4	10.8	NA	NA	NA	NA	NA
SMU_1787c	Secreted protein	T95	0.94	10.1	9.9	9.5	NA	NA	NA	NA	NA	NA	NA
SMU_1879	PTS system, mannose-specific component IID	S174	1.00	11.4	10.3	11.0	11.1	NA	NA	NA	NA	NA	NA
SMU_1936c	DNA-directed RNA polymerase subunit delta	S128	0.99	10.1	10.8	10.5	10.3	NA	NA	NA	NA	NA	NA
SMU_205c	Bacteriocin immunity protein	S64	1.00	11.6	11.5	11.4	11.1	NA	NA	NA	NA	NA	NA
SMU_393	Regulator of chromosome segregation-like C-terminal domain-containing protein	T130	1.00	10.1	10.5	10.2	10.3	13.3	NA	NA	NA	NA	NA
SMU_501	Uncharacterized protein	Y70	1.00	10.6	10.2	9.2	10.4	NA	NA	NA	NA	NA	NA
SMU_502	TcaA 4th domain-containing protein	S53	0.87	11.3	11.3	11.1	11.1	13.3	NA	NA	NA	NA	NA
SMU_793	Thioesterase domain-containing protein	T84	1.00	10.0	10.9	10.9	10.7	NA	NA	NA	NA	NA	NA

*Localization probability (LP); indicates the confidence that phosphorylation occurred at that specific amino acid residue

Table S7 Transcriptomics comparison of *S. mutans* CRISRPi^{gpsB} without and with 0.5% xylose

Gene	log2FC	FDR
cas9	7.88	1.32E-166
SMU_984	3.08	6.05E-60
SMU_503c	3.02	4.23E-34
SMU_609	2.99	1.47E-11
SMU_791c	2.94	8.16E-11
SMU_566c	2.62	2.47E-02
SMU_1402c	2.60	1.22E-17
SMU_189	2.51	1.01E-04
SMU_935	2.46	1.89E-03
leuD	2.32	3.52E-39
SMU_18	2.32	1.27E-05
SMU_1403c	2.21	2.05E-13
SMU_238c	2.17	8.96E-18
SMU_930c	2.16	7.95E-14
leuC	2.14	2.15E-59
SMU_215c	2.11	1.68E-03
SMU_115	2.11	9.32E-03
SMU_1404c	2.08	2.25E-13
SMU_216c	2.03	1.45E-03
SMU_934	2.02	1.53E-04
SMU_237c	2.01	1.62E-17
SMU_1928	-2.00	2.78E-06
xis	-2.07	3.50E-01
SMU_41	-2.09	1.79E-01
opuAa	-2.10	9.15E-84

SMU_1395c	-2.13	4.50E-10
SMU_472	-2.33	1.85E-48
gbpC	-2.36	1.32E-21
SMU_2146c	-2.46	4.03E-57
SMU_471	-5.26	6.55E-146

Table S8 Proteomics comparison of *S. mutans* CRISRPi^{gpsB} without and with 0.5% xylose

Gene	Description	log2FC	adj.P.Val
cas9	CRISPR-associated endonuclease Cas9	7.35	5.16E-08
treA	Alpha,alpha-phosphotrehalase	3.24	5.31E-06
pttB	PTS system, trehalose-specific IIABC component	2.81	2.84E-05
lacD2	Tagatose 1,6-diphosphate aldolase 2	2.31	9.23E-04
SMU_1335c	Enoyl-(Acyl-carrier-protein) reductase	2.01	2.01E-04
SMU_936	Amino acid ABC transporter, ATP-binding protein	1.92	1.63E-02
SMU_933	Amino acid ABC transporter, periplasmic amino acid-binding protein	1.76	1.49E-02
radC	DNA repair protein RadC	1.72	2.25E-01
SMU_932	Uroporphyrinogen decarboxylase (URO-D) domain-containing protein	1.64	1.32E-02
sodA	Superoxide dismutase [Mn/Fe]	1.49	1.35E-03
SMU_1196c	DUF3862 domain-containing protein	1.48	1.61E-05
SMU_961	Carboxymuconolactone decarboxylase-like domain-containing protein	1.41	4.60E-03
SMU_1166c	ABC transporter, permease protein	1.37	4.98E-01
SMU_237c	Integral membrane protein	1.31	2.01E-04
SMU_1068c	ABC transporter, ATP-binding protein	1.31	3.27E-03
SMU_2057c	Cd(2+)-exporting ATPase	1.31	6.75E-03
SMU_1217c	ABC transporter, amino acid binding protein	1.31	1.64E-05
gcrR	Response regulator GcrR for glucan-binding protein C	1.30	3.52E-05
priA	Primosomal protein N	1.28	3.11E-06
SMU_1367c	Methyltransferase domain-containing protein	1.25	3.75E-03
SMU_896	Uncharacterized protein	1.23	1.77E-04
SMU_633	Thioesterase	1.21	6.44E-05
apbE	FAD:protein FMN transferase	1.21	7.29E-06

metE	5-methyltetrahydropteroyltriglutamate--homocysteine methyltransferase	1.20	1.92E-04
aldB	Alpha-acetolactate decarboxylase	1.19	3.71E-05
SMU_643	Esterase	1.14	2.45E-03
glyA	Serine hydroxymethyltransferase	1.13	3.91E-06
SMU_1067c	ABC transporter, permease protein	1.13	2.43E-04
rnz	Ribonuclease Z	1.10	4.93E-05
SMU_998	ABC transporter, periplasmic ferrichrome-binding protein	1.10	1.22E-03
SMU_2027	Transcriptional regulator	1.08	2.73E-04
SMU_386	[Ribosomal protein bS18]-alanine N-acetyltransferase	1.08	4.87E-04
SMU_874	Hcy-binding domain-containing protein	1.08	1.40E-03
SMU_1957	PTS system, mannose-specific IID component	1.06	1.06E-03
SMU_296	Ketopantoate reductase N-terminal domain-containing protein	1.06	5.78E-03
SMU_1070c	HTH LytTR-type domain-containing protein	1.05	1.09E-02
rplS	Large ribosomal subunit protein bL19	1.05	1.19E-04
leuA	2-isopropylmalate synthase	1.04	5.09E-03
SMU_1487	Cobalamin-independent methionine synthase MetE C-terminal/archaeal domain-containing protein	1.03	7.25E-05
nagB	Glucosamine-6-phosphate deaminase	1.03	4.93E-05
SMU_1284c	NAD(P)-binding domain-containing protein	1.03	1.50E-02
SMU_1046c	GTP pyrophosphokinase	1.02	3.09E-04
ogt	Methylated-DNA--protein-cysteine methyltransferase	1.02	1.19E-04
rpsU	Small ribosomal subunit protein bS21	1.02	1.17E-03
SMU_954	pyridoxal kinase	1.02	1.84E-04
SMU_679	Oxidoreductase, aldo/keto reductase family	1.01	2.09E-04
cshA	DEAD-box ATP-dependent RNA helicase CshA	1.01	1.89E-05

adhA	Acetoin dehydrogenase (TPP-dependent), E1 component alpha subunit	1.00	3.83E-04
SMU_220c	Uncharacterized protein	1.00	4.61E-05
SMU_820	HlyD family secretion protein	1.00	1.25E-02
urdA	Urocanate reductase	-1.01	5.05E-05
SMU_1976c	Uncharacterized protein	-1.01	1.77E-02
SMU_1956c	PTS fructose transporter subunit IA	-1.01	1.88E-03
SMU_646	Phosphatase	-1.02	4.06E-03
SMU_1730c	Acetyltransferase	-1.03	4.50E-04
tig	Trigger factor	-1.03	7.84E-04
pheT	Phenylalanine--tRNA ligase beta subunit	-1.03	1.57E-05
SMU_275	L-ribulose-5-phosphate 4-epimerase	-1.03	4.30E-05
SMU_36	Suppressor of fused-like domain-containing protein	-1.03	2.39E-03
hisH	Imidazole glycerol phosphate synthase subunit HisH	-1.04	1.12E-04
gltB	NADPH-dependent glutamate synthase (Small subunit)	-1.04	8.45E-04
SMU_796	NmrA-like domain-containing protein	-1.05	5.47E-06
hisF	Imidazole glycerol phosphate synthase subunit HisF	-1.05	4.80E-04
SMU_794	Uncharacterized protein	-1.06	4.02E-05
SMU_564	DUF1797 family protein	-1.06	2.09E-02
SMU_303	Metallo-beta-lactamase domain-containing protein	-1.07	3.38E-04
SMU_1587c	AAA family ATPase	-1.07	3.12E-03
SMU_100	Sorbose PTS system, IIB component	-1.08	1.34E-05
SMU_2054c	DUF3013 domain-containing protein	-1.10	1.18E-02
hisC	Histidinol-phosphate aminotransferase	-1.11	8.32E-05
fruI	Inducible fructose permease	-1.11	1.11E-04
serB	phosphoserine phosphatase	-1.11	3.65E-04
SMU_172	SpoVT-AbrB domain-containing protein	-1.11	7.36E-04

hisG	ATP phosphoribosyltransferase	-1.12	2.48E-03
SMU_322c	UTP--glucose-1-phosphate uridylyltransferase	-1.12	3.86E-05
SMU_673	ABC transporter permease	-1.12	4.84E-03
SMU_405c	Transcriptional regulator	-1.13	1.06E-03
SMU_494	Transaldolase	-1.15	1.68E-05
ybeY	Endoribonuclease YbeY	-1.16	1.22E-03
gatC	Aspartyl/glutamyl-tRNA(Asn/Gln) amidotransferase subunit C	-1.17	7.91E-03
rlmH	Ribosomal RNA large subunit methyltransferase H	-1.17	1.06E-03
hisA	1-(5-phosphoribosyl)-5-[(5-phosphoribosylamino)methylideneamino] imidazole-4-carboxamide isomerase	-1.20	2.01E-04
SMU_1633c	Foldase	-1.23	3.34E-04
opuAa	Quaternary amine transport ATP-binding protein	-1.23	8.58E-05
groEL	Chaperonin GroEL	-1.23	1.64E-05
gltA	Glutamate synthase (Large subunit)	-1.24	3.08E-04
SMU_776	Methyltransferase	-1.25	5.29E-03
hisZ	ATP phosphoribosyltransferase regulatory subunit	-1.27	3.52E-05
pheS	Phenylalanine--tRNA ligase alpha subunit	-1.27	5.31E-06
SMU_44	Histidine kinase/HSP90-like ATPase domain-containing protein	-1.29	6.69E-04
rnpA	Ribonuclease P protein component	-1.29	1.21E-03
SMU_1641c	CsbD-like domain-containing protein	-1.30	5.64E-03
SMU_1588c	Hexosyltransferase	-1.30	3.11E-06
SMU_470	UPF0398 protein SMU_470	-1.31	2.95E-04
SMU_690	DUF6287 domain-containing protein	-1.31	4.72E-04
purA	Adenylosuccinate synthetase	-1.31	3.50E-06
SMU_769	Transcriptional coactivator p15 (PC4) C-terminal domain-containing protein	-1.32	1.72E-02

sloA	ABC transporter, ATP-binding protein possible iron and/or manganese ABC transport system	-1.33	5.58E-03
SMU_1776c	DNA-damage-inducible protein J	-1.34	4.87E-04
SMU_1511c	Acetyltransferase	-1.35	1.39E-04
acpP	Acyl carrier protein	-1.36	2.63E-03
glgC	Glucose-1-phosphate adenylyltransferase	-1.37	2.69E-05
glmS	Glutamine--fructose-6-phosphate aminotransferase [isomerizing]	-1.43	1.50E-05
hisD	Histidinol dehydrogenase	-1.45	1.09E-04
SMU_392c	Uncharacterized protein	-1.50	1.08E-03
SMU_312	PTS system, sorbitol phosphotransferase enzyme IIBC	-1.50	6.49E-03
SMU_843	Capsule synthesis protein CapA domain-containing protein	-1.57	2.07E-04
SMU_409	tRNA threonylcarbamoyladenosine biosynthesis protein TsaE	-1.59	1.44E-04
SMU_694c	Ferredoxin (4Fe-4S)	-1.60	1.65E-03
ptxA	Ascorbate-specific PTS system EIIA component	-1.60	6.70E-05
SMU_1400c	HicB-like antitoxin of toxin-antitoxin system domain-containing protein	-1.62	1.53E-04
rpiB	Sugar-phosphate isomerase	-1.63	3.14E-03
gbpA	Glucan-binding protein A, GbpA	-1.67	4.30E-03
SMU_531	Chorismate mutase	-1.69	3.11E-03
dltC	D-alanyl carrier protein	-1.69	3.83E-04
SMU_371	ABC-2 type transporter transmembrane domain-containing protein	-1.70	1.04E-04
copY	Negative transcriptional regulator, CopY	-1.72	9.32E-05
comYD	Competence protein ComYD	-1.74	2.31E-02
SMU_797	DUF4649 domain-containing protein	-1.75	3.24E-04
SMU_1723c	HD domain-containing protein	-1.83	2.45E-05
dinB	DNA polymerase IV	-1.85	1.50E-05

pdhA	Pyruvate dehydrogenase, TPP-dependent E1 component alpha-subunit	-1.86	5.67E-03
pfl	Formate acetyltransferase	-1.95	4.47E-06
SMU_1724c	rRNA methylase	-2.03	8.53E-05
psaR	Transcriptional regulator	-2.05	2.05E-03
SMU_472	THUMP domain-containing protein	-2.07	5.16E-08
SMU_308	Sorbitol-6-phosphate 2-dehydrogenase	-2.09	2.27E-03
SMU_689	Lysozyme	-2.13	2.71E-04
fruA	Fructan beta-fructosidase	-2.19	6.99E-05
SMU_174c	Polymerase nucleotidyl transferase domain-containing protein	-2.22	4.80E-04
SMU_609	40K cell wall protein	-2.24	1.54E-02
SMU_1981c	Competence protein ComGF	-2.26	1.05E-01
wapE	Gram-positive cocci surface proteins LPxTG domain-containing protein	-2.29	5.35E-04
acp	Acyl carrier protein	-2.29	1.89E-05
SMU_1256c	Phage protein	-2.31	6.34E-05
glnA	Glutamate--ammonia ligase	-2.31	8.18E-06
SMU_151	Bacteriocin	-2.33	5.48E-04
rpoE	Probable DNA-directed RNA polymerase subunit delta	-2.34	2.01E-04
dexA	Dextranase	-2.34	1.12E-04
wapA	Wall-associated protein	-2.39	4.94E-04
acn	Aconitate hydratase A	-2.40	2.87E-03
adhE	Aldehyde-alcohol dehydrogenase	-2.45	1.92E-06
spaP	Cell surface antigen I/II	-2.48	2.29E-04
SMU_11	Recombinase	-2.50	4.35E-04
pdhC	Dihydrolipoamide acetyltransferase component of pyruvate dehydrogenase complex	-2.51	5.39E-03
flaW	Flavodoxin	-2.52	1.42E-04

citZ	Citrate synthase	-2.53	3.72E-03
xseB	Exodeoxyribonuclease 7 small subunit	-2.54	3.99E-04
gtfC	Glucosyltransferase-SI	-2.76	2.07E-04
comYB	ABC transporter ComYB probably part of the DNA transport machinery	-2.82	1.33E-02
SMU_1904c	Bacteriocin transport accessory protein	-2.90	1.91E-03
comEA	Competence protein	-2.93	2.58E-02
SMU_626	Competence protein	-2.99	1.68E-03
SMU_2147c	LysM domain-containing protein	-3.14	7.05E-03
gbpC	Glucan-binding protein C, GbpC	-3.39	1.12E-04
icd	Isocitrate dehydrogenase [NADP]	-3.42	7.98E-04
gpsB	Cell cycle protein GpsB	-3.89	3.40E-07
comX1	ComX1, transcriptional regulator of competence-specific genes	-3.92	4.55E-03
smf	DNA processing Smf protein	-4.19	2.33E-02
comYA	ABC transporter, ATP-binding protein ComYA late competence protein	-4.21	1.42E-02
SMU_370	ABC transporter, ATP-binding protein	-4.34	9.40E-10
SMU_1657c	Nitrogen regulatory protein PII	-4.65	2.44E-03
SMU_1327c	4Fe-4S ferredoxin-type domain-containing protein	-4.75	1.05E-05
SMU_372	Uncharacterized protein	-4.82	1.61E-05
gtfB	Glucosyltransferase-I	-4.90	1.50E-05
ssb2	Single-stranded DNA-binding protein	-5.21	1.42E-02
SMU_373	Methyltransferase type 11 domain-containing protein	-5.21	6.85E-10
SMU_376	Aminotransferase	-6.20	3.40E-07
comYC	Competence protein ComYC	-6.60	6.25E-03
SMU_836	Peptidase C51 domain-containing protein	-7.48	6.91E-03
SMU_374	Oxidoreductase	-8.25	2.70E-06

Table S9 Phosphoproteomics comparison of *S. mutans* P_{xyl}-d*cas9* sgRNA^{gpsB} vs *S. mutans* P_{xyl}-d*cas9* sgRNA^{gpsB} with 0.5% xylose

Gene	Description	Phosphorylation site	Phospho logFC	adj.P.Val	Protein logFC
asd	Aspartate-semialdehyde dehydrogenase	S96	0.97	0.14	-0.33
cas9	CRISPR-associated endonuclease Cas9	S793	PRESENT	NA	7.34
		S804	PRESENT	NA	7.34
		S960	PRESENT	NA	7.34
		S1085	PRESENT	NA	7.34
divIVA	Cell division protein DivIVA	T226	-3.22	0.00	-0.56
		T232	-2.07	0.00	-0.56
		T195	-2.00	0.00	-0.56
		T77	-1.32	0.01	-0.56
		T90	-1.03	0.14	-0.56
		T201	-0.87	0.01	-0.56
		T262	0.34	0.26	-0.56
		S70	ABSENT	NA	-0.56
ffh	Signal recognition particle protein	S397	0.57	0.23	-0.31
gatC	Aspartyl/glutamyl-tRNA(Asn/Gln) amidotransferase subunit C	S67	ABSENT	NA	-1.17
glnA	Glutamate--ammonia ligase	Y125	0.49	0.25	-2.32
glyA	Serine hydroxymethyltransferase	Y55	2.34	0.01	1.14
gpsB	Cell cycle protein GpsB	T88	-1.22	0.05	-3.89
		T73	-0.82	0.05	-3.89
		T87	ABSENT	NA	-3.89
gtfB	Glucosyltransferase-I	T1041	ABSENT	NA	-4.91
		T119	ABSENT	NA	-4.91
		T121	ABSENT	NA	-4.91
mapZ	Mid-cell-anchored protein Z	T127	-1.58	0.01	-0.55

		S125	-0.85	0.02	-0.55
mltG	Endolytic murein transglycosylase	T191	-1.51	0.14	-0.32
		T166	-0.45	0.24	-0.32
		T105	0.52	0.27	-0.32
murD	UDP-N-acetylmuramoylalanine--D-glutamate ligase	S331	-1.09	0.06	0.04
pgi	Glucose-6-phosphate isomerase	T149	1.53	0.02	0.12
pknB	non-specific serine/threonine protein kinase	T291	0.62	0.40	0.05
ptcC	Permease IIC component	Y437	0.76	0.07	-0.24
ptsG	PTS system, glucose-specific IIABC component	T507	0.94	0.02	0.23
ptsH	Phosphocarrier protein HPr	S31	-0.87	0.13	-0.56
		T12	-0.75	0.14	-0.56
pttB	PTS system, trehalose-specific IIABC component	S491	2.76	0.00	2.79
		T485	3.26	0.00	2.79
		T487	3.65	0.00	2.79
		S493	PRESENT	NA	2.79
pyrH	Uridylate kinase	S14	PRESENT	NA	-0.41
rplJ	Large ribosomal subunit protein uL10	S2	0.96	0.14	0.40
rplO	Large ribosomal subunit protein uL15	S131	-0.33	0.48	0.62
rplU	Large ribosomal subunit protein bL21	Y4	PRESENT	NA	0.62
rpmF	Large ribosomal subunit protein bL32	T32	ABSENT	0.79	0.23
		T33	ABSENT	NA	0.23
rpoY	DNA-directed RNA polymerase subunit epsilon	T74	-0.30	0.63	-0.56
rpsE	Small ribosomal subunit protein uS5	S159	1.07	0.01	0.64
rs1	Ribosomal protein S1 sequence specific DNA-binding protein	T198	-0.10	0.83	0.46
SMU_1080c	CwlT-like lysozyme domain-containing protein	T47	1.22	0.01	0.26
SMU_1633c	Foldase	T7	-0.92	0.06	-1.28
	CsbD-like domain-containing protein	T29	-1.96	0.00	-1.29

SMU_1641c		S60	-1.54	0.01	-1.29
SMU_1702c	Phosphatase	Y7	0.61	0.41	ABSENT
SMU_1772c	Uncharacterized protein	T56	1.35	0.24	-0.10
SMU_1787c	Secreted protein	T95	-0.59	0.29	-0.58
SMU_1896c	Uncharacterized protein	Y16	PRESENT	NA	ABSENT
pgfM2	Transmembrane protein	T16	-2.97	0.00	-1.04
SMU_2079c	UPF0297 protein SMU_2079c	T7	0.26	0.35	-0.57
		T4	0.94	0.01	-0.57
SMU_2152c	Cytoskeleton protein RodZ-like C-terminal domain-containing protein	S77	-1.04	0.09	-0.31
SMU_447	UPF0291 protein SMU_447	T62	0.14	0.79	-0.87
SMU_502	FecR protein domain-containing protein	S53	1.27	0.00	-0.71
		T52	1.46	0.00	-0.71
SMU_564	DUF1797 family protein	S19	ABSENT	NA	-1.05
SMU_668c	Ribonucleoside-diphosphate reductase	S157	3.06	0.00	0.71
SMU_706c	Proton-coupled thiamine transporter YuaJ	Y10	PRESENT	NA	ABSENT
SMU_757	Gas vesicle protein	T76	-0.25	0.63	-0.55
tig	Trigger factor	S2	-0.96	0.05	-1.04
tsf	Elongation factor Ts	T5	-0.59	0.30	-0.06
tuf	Elongation factor Tu	S52	0.24	0.75	0.26

Table S10 Phosphoproteomics comparison of *S. mutans* UA159 vs *S. mutans* Δ*gpsB*$_{sup}$

Gene	Description	Phosphorylation site	Phospho logFC	adj.P.Val	Protein logFC
cas1	CRISPR-associated endonuclease Cas1	S15	1.18	0.00	ABSENT
		Y16	1.18	0.00	ABSENT
clpP	ATP-dependent Clp protease proteolytic subunit	S10	0.92	0.01	0.65
coaD	Phosphopantetheine adenylyltransferase	T117	0.60	0.04	-0.09
divIVA	Cell division protein DivIVA	T77	ABSENT	NA	0.18
		T262	4.21	0.01	0.18
		T201	3.01	0.02	0.18
		T195	1.72	0.03	0.18
ffh	Signal recognition particle protein	S397	-0.59	0.01	0.23
frr	Ribosome-recycling factor	S19	-1.09	0.00	-0.58
fruA	Fructan beta-fructosidase	S1355	0.81	0.01	-2.84
		S1356	0.81	0.01	-2.84
		S1355	0.61	0.01	-2.84
		S1356	0.61	0.01	-2.84
		S1358	0.61	0.01	-2.84
fruI	Inducible fructose permease	T57	0.26	0.50	0.52
		S291	-0.06	0.88	0.52
		S30	-0.80	0.02	0.52
fusA	Elongation factor G	S638	-0.51	0.09	-0.14
gapC	Glyceraldehyde-3-phosphate dehydrogenase	S212	0.33	0.25	0.00
glnQ	Amino acid ABC transporter, ATP-binding protein	T247	2.68	0.00	0.36
gpsB	Cell cycle protein GpsB	T72	ABSENT	NA	-7.23
groEL	Chaperonin GroEL	S460	1.64	0.00	0.27
		S356	0.23	0.44	0.27
		S349	-0.14	0.70	0.27
lepA	Elongation factor 4	T39	-0.33	0.37	0.03
mapZ	Mid-cell-anchored protein Z	T127	-2.55	0.00	0.24

		T18	-4.46	0.00	0.24
mecA	Adapter protein MecA	S57	-0.81	0.12	-0.20
mltG	Endolytic murein transglycosylase	T102	ABSENT	NA	-0.08
		T166	-0.74	0.38	-0.08
		T191	-5.94	0.00	-0.08
		T105	-6.00	0.00	-0.08
pfkA	ATP-dependent 6-phosphofructokinase	S159	0.77	0.26	0.17
pgi	Glucose-6-phosphate isomerase	T149	1.48	0.01	0.10
pknB	non-specific serine/threonine protein kinase	T291	-0.10	0.82	0.23
ppc	Phosphoenolpyruvate carboxylase	S719	-2.10	0.00	0.55
ptcC	Permease IIC component	Y437	1.72	0.00	0.45
ptnA	PTS system mannose-specific EIIAB component	T203	PRESENT	NA	-0.20
ptsH	Phosphocarrier protein HPr	T12	-0.23	0.47	-3.49
pttB	PTS system, trehalose-specific IIABC component	T487	0.31	0.15	0.38
		T485	-0.30	0.12	0.38
		S493	-0.31	0.16	0.38
		S491	-0.60	0.01	0.38
rplJ	Large ribosomal subunit protein uL10	S2	3.07	0.01	0.52
rplO	Large ribosomal subunit protein uL15	S131	-1.33	0.00	-0.19
rplQ	Large ribosomal subunit protein bL17	S74	0.50	0.22	0.25
rplR	Large ribosomal subunit protein uL18	S89	-0.83	0.03	0.17
rplW	Large ribosomal subunit protein uL23	Y4	PRESENT	NA	-0.01
rpmF	Large ribosomal subunit protein bL32	S37	0.76	0.01	-0.31
		T32	-3.42	0.00	-0.31
rpsE	Small ribosomal subunit protein uS5	S154	-0.71	0.00	-0.07
		S159	-0.86	0.00	-0.07
rpsM	Small ribosomal subunit protein uS13	S50	0.30	0.16	0.47
rpsO	Small ribosomal subunit protein uS15	T52	0.98	0.02	0.35
		Y53	0.98	0.02	0.35
rs1		S123	0.04	0.94	0.43

	Ribosomal protein S1 sequence specific DNA-binding protein	T198	-0.93	0.01	0.43
scnK	histidine kinase	T257	1.81	0.00	0.38
SMU_1080c	CwlT-like lysozyme domain-containing protein	T47	0.07	0.82	0.03
SMU_1143c	Riboflavin biosynthesis protein	T245	0.87	0.03	0.52
SMU_1157c	SIR2-like domain-containing protein	S80	1.68	0.01	0.49
		S83	1.68	0.01	0.49
SMU_1367c	Methyltransferase domain-containing protein	Y28	-2.50	0.00	-0.09
SMU_160	DUF2268 domain-containing protein	S177	-1.74	0.01	PRESENT
		Y168	-1.74	0.01	PRESENT
SMU_1621c	UPF0346 protein SMU_1621c	T11	-0.22	0.33	-0.73
		Y6	-0.22	0.33	-0.73
SMU_1772c	Uncharacterized protein	Y55	1.54	0.01	-0.43
		T56	1.33	0.02	-0.43
SMU_1787c	Secreted protein	S123	-0.81	0.01	-0.34
SMU_1896c	Uncharacterized protein	Y16	1.73	0.00	ABSENT
levX	PTS fructose transporter subunit IA	Y11	-2.40	0.01	-0.83
levF	PTS system, mannose-specific IIC component	S269	-1.57	0.00	-1.06
levE	PTS system, mannose-specific IIB component	T17	-0.59	0.10	-2.27
		T19	-0.71	0.03	-2.27
levD	PTS system, sugar-specific enzyme IIA component	S135	-1.21	0.00	-2.44
pgfM2	Transmembrane protein	T16	-3.47	0.00	-0.29
ireB-like	UPF0297 protein SMU_2079c	T7	4.85	0.01	0.28
		T4	-5.80	0.00	0.28
SMU_2123	Uncharacterized protein	Y82	PRESENT	NA	ABSENT

SMU_447	UPF0291 protein SMU_447	T62	-3.67	0.00	-0.11
SMU_470	UPF0398 protein SMU_470	Y144	-0.40	0.34	0.07
		Y146	-0.40	0.34	0.07
SMU_502	TcaA 4th domain-containing protein	S53	1.69	0.04	0.01
		T52	1.58	0.04	0.01
SMU_528c	ABM domain-containing protein	S83	0.24	0.53	-1.18
SMU_54	Amino acid recemase	T128	-1.23	0.01	-0.39
		Y127	-1.23	0.01	-0.39
SMU_632	Transcriptional regulator	S67	1.20	0.01	ABSENT
		T70	1.20	0.01	ABSENT
SMU_635	Integral membrane protein	S103	1.45	0.00	0.68
SMU_668c	Ribonucleoside-diphosphate reductase	S157	1.06	0.14	1.00
SMU_695	LysM domain-containing protein	T14	-1.22	0.03	0.29
SMU_739c	Adhesin	T472	1.03	0.05	0.00
SMU_757	Gas vesicle protein	T76	-3.81	0.00	-0.77
SMU_874	Hcy-binding domain-containing protein	S480	0.37	0.13	ABSENT
		T482	0.37	0.13	ABSENT
ssb	Single-stranded DNA-binding protein	T12	-2.65	0.00	0.07
tsf	Elongation factor Ts	T5	4.64	0.00	-0.48
tuf	Elongation factor Tu	Y50	-0.16	0.77	-0.48
		T387	-1.33	0.00	-0.48
		S52	-1.83	0.00	-0.48
valS	Valine--tRNA ligase	S6	1.37	0.00	-0.07

Table S11 Strains used in this study

Strain	Description	Source
S. mutans strains		
UA159	Wild-type	Shields Lab
Δ*pknB*	*pknB*::*aphA3*, kanamycin resistant	(1)
Δ*pppL*	*pppL*::*aphA3*, kanamycin resistant	(1)
Δ*gpsB*$_{sup}$	*gpsB*::*aphA3*, kanamycin resistant; SMU_470 (N154S), *pppL* (G98R), SMU_1232c (S42R)	This study
CRISPRi^{gpsB}	Δ*cas9* AT>TA (start codon to stop codon mutation); pPM::sgRNA-*gpsB*; P*xyl*-d*cas*9$_{Smu}$; kanamycin and spectinomycin resistant	(2)

References

1. Shields RC, O'Brien G, Maricic N, Kesterson A, Grace M, Hagen SJ, Burne RA. 2018. Genome-wide screens reveal new gene products that influence genetic competence in *Streptococcus mutans*. J Bacteriol 200:16 e00508-17.

2. Shields RC, Walker AR, Maricic N, Chakraborty B, Underhill SAM, Burne RA. 2020. Repurposing the *Streptococcus mutans* CRISPR-Cas9 System to Understand Essential Gene Function. PLoS Pathog 16:e1008344.
